# Bromotyrosine-derived alkaloids from the Caribbean sponge *Aplysina lacunosa*

**DOI:** 10.3762/bjoc.11.254

**Published:** 2015-11-26

**Authors:** Qun Göthel, Thanchanok Sirirak, Matthias Köck

**Affiliations:** 1 Alfred-Wegener-Institut, Helmholtz-Zentrum für Polar- und Meeresforschung, Am Handelshafen 12, 27570 Bremerhaven, Germany

**Keywords:** alkaloids, *Aplysina lacunosa*, bromotyrosine, marine natural products, NMR spectroscopy

## Abstract

Three new bromotyrosine-derived alkaloids 14-debromo-11-deoxyfistularin-3 (**1**), aplysinin A (**2**), and aplysinin B (**3**), together with 15 known compounds (**4**–**18**) were isolated from the sponge *Aplysina lacunosa* collected from Stirrup Cay, Bahamas. The structures of the isolated compounds were identified on the basis of MS and NMR data analysis. The ^13^C NMR assignment of spirocyclohexadienylisoxazoline moieties of **1** and **2** were confirmed by an 1,1-ADEQUATE experiment. Compounds **1** and **2** showed a mild to moderate cytotoxic activities against KB-31 and FS4-LTM cell lines. Only aplysinin A (**2**) exhibited cytotoxicity against MCF-7 cells.

## Introduction

**Figure 1:**
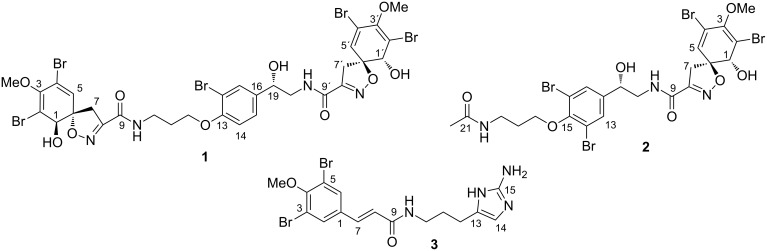
Three new bromotyrosine derivatives isolated from sponge *Aplysina lacunosa*: 14-debromo-11-deoxyfistularin-3 (**1**), aplysinin A (**2**), and aplysinin B (**3**).

Bromotyrosine-derived alkaloids are unique brominated metabolites which were isolated mainly from marine sponges of the order Verongida. For more than 50 years, bromotyrosine alkaloids raised the interests of synthetic and natural products chemists due to their high chemical diversity (besides the common spirocyclohexadienylisoxazoline moiety) and interesting biological activities. Since the first derivative 2,6-dibromo-4-acetamido-4-hydroxycyclohexadienone was isolated in 1967 [1], a series of bromotyrosine alkaloids were discovered with various biological activities, including antiviral [2], antibiotic [3–5], Na^+^/K^+^ ATPase inhibition [6–8], anti-HIV [9–10], antifungal [11], histidine-H3 antagonist [12], cytotoxic [13–14], and antimalarial activities [15–17]. During our investigation of the chemical constituents of *Aplysina lacunosa* (Aplysinidae, Verongida), three new bromotyrosine-derived alkaloids: 14-debromo-11-deoxyfistularin-3 (**1**), aplysinin A (**2**), and aplysinin B (**3**) (Figure 1), together with 15 known compounds were obtained. In this report we describe the structure elucidation of **1** to **3** and the biological activities of all the isolated compounds.

## Results and Discussion

**Figure 2:**
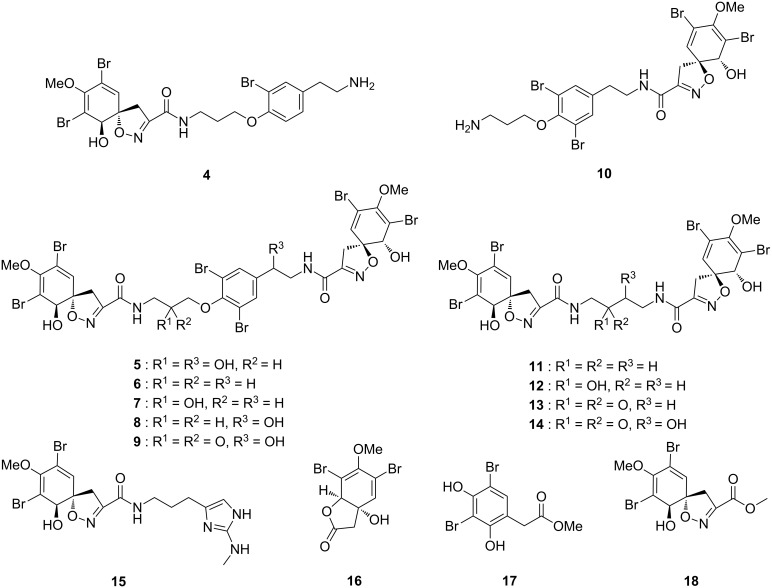
Bromotyrosine alkaloids and brominated compounds isolated from the sponge *Aplysina lacunosa*.

The freeze-dried sponge was extracted three times with CH_2_Cl_2_/MeOH (1:1, v/v). The resulting crude extract was partitioned between *n*-hexane and MeOH. The MeOH extract was further partitioned between ethyl acetate and H_2_O. The resulting ethyl acetate phase was purified by vacuum liquid chromatography using silica gel with a stepwise gradient eluent from 100:0 to 80:20 (CH_2_Cl_2_/MeOH, v/v). The two fractions eluting with 97:3 and 94:6 (CH_2_Cl_2_/MeOH, v/v) were further purified by HPLC and yielded the three new bromotyrosine derivatives 14-debromo-11-deoxyfistularin-3 (**1**), aplysinins A (**2**) and B (**3**), as well as the 15 known compounds: 14-debromoaraplysillin I (**4**) [18], fistularin-3 (**5**) [19], 11,19-dideoxyfistularin-3 (**6**) [20], 19-deoxyfistularin-3 (**7**) [21], 11-deoxyfistularin-3 (**8**) [22], 11-ketofistularin-3 (**9**) [2], hexadellin B (**10**) [23], aerothionin (**11**) [13,24–25], 11-hydroxyaerothionin (**12**) [20], 11-oxoaerothionin (**13**) [13], 11-oxo-12-hydroxyaerothionin (**14**) [26], *N*-methyl-aerophobin-2 (**15**) [27], aeroplysinin-2 (**16**) [28], subereaphenol B (**17**) [29], and the unnamed bromotyrosine **18** [30]. Compounds **4** to **18** were identified by comparison of their MS data as well as ^1^H and ^13^C NMR chemical shifts with those reported in the literature (Figure 2).

**Figure 3:**
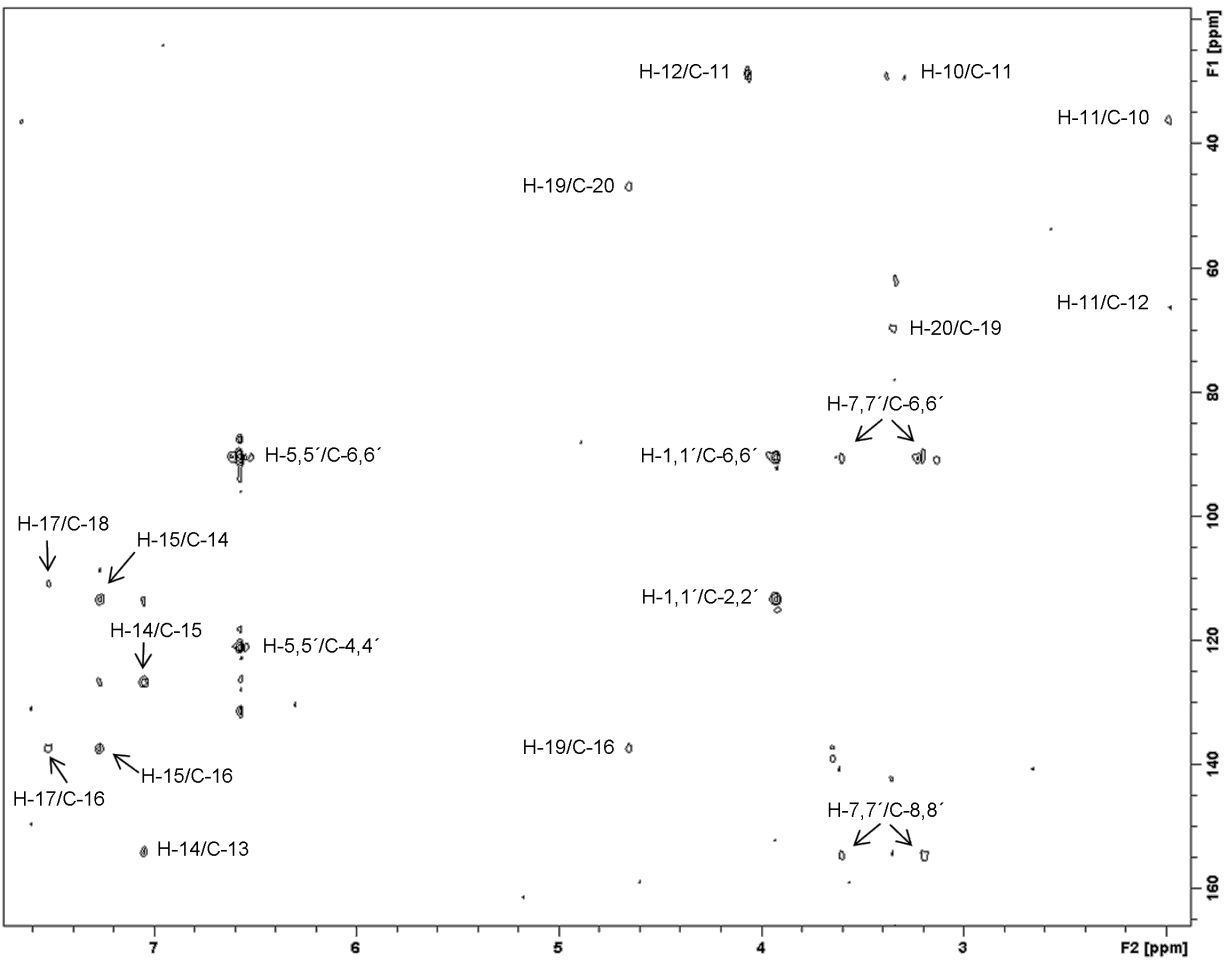
1,1-ADEQUATE spectrum of 14-debromo-11-deoxyfistularin-3 (**1**).

Compound **1** was obtained as a white solid. The MS–ESI(+) showed a characteristic pentabrominated ion peak cluster at *m/z* 1037/1039/1041/1043/1045/1047 [M + Na]^+^ (1:5:10:10:5:1). The molecular formula of C_31_H_31_Br_5_N_4_O_10_ was deduced from HRMS–ESI(+) at *m/z* 1036.7844 [M + Na]^+^ (calcd for C_31_H_31_^79^Br_5_N_4_O_10_Na, 1036.7855) which required 16 double bond equivalents (DBEs). The ^13^C NMR spectrum of **1** indicated two amide groups at δ 158.9 (C-9´) and 159.5 (C-9), 14 olefinic carbons, two hetero-olefinic carbons at δ 154.5 (C-8´) and 155.0 (C-8), and 5 ring systems to fulfill the DBEs. The comparison of the ^1^H and ^13^C NMR data from positions C-1, C-1´ to C-9, C-9´(Table 1) of **1** with those of aerothionin (**11**) [13,24–25] allowed the assignment of the two dibromospirocyclohexadienylisoxazole carbonyl groups which was further confirmed by ^1^H,^13^C-HMBC and 1,1-ADEQUATE experiments. The ^13^C NMR assignment of C-2 and C-4 were reversed before Ciminiello´s revision in 1994 [26]. Nevertheless, the wrong assignment has still continued to be used as reference in the literature [29,31]. We therefore applied an 1,1-ADEQUATE experiment which allows the selective observation of two-bond H,C correlations [32]. The signals in the 1,1-ADEQUATE spectrum from δ_H_ 3.92 (H-1, H-1´) to δ_C_ 113.6 (C-2, C-2´) and from δ_H_ 6.57 (H-5, H-5´) to δ_C_ 120.9 and 120.8 (C-4 and C-4´, respectively) confirmed the assignments of C-2, C-2´ and C-4, C-4´ from 1994 (Figure 3). ^1^H,^1^H-COSY correlations were observed among 9-NH (δ 8.60)/H-10 (δ 3.36, 2H)/H-11 (δ 1.95, 2H)/H-12 (δ 4.06, 2H) establishing an propanamine moiety. In a similar manner, ^1^H,^1^H-COSY correlations among 9´-NH (δ 8.35)/H-20 (δ 3.35)/H-19 (δ 4.65, 2H) revealed the presence of a hydroxylethylamine moiety. The remaining signals at δ_H_ 7.05 (d, *J* = 8.6 Hz, H-14), 7.28 (dd, *J* = 1.5, 8.6 Hz, H-15), and 7.52 (d, *J* = 1.5 Hz, H-17) together with ^1^H,^13^C-HMBC correlations indicated the presence of a 1,2,4-trisubstituted phenoxy group. The phenoxy group was connected to the propanamine and the hydroxyethylamine substructures according to ^1^H,^13^C-HMBC correlations from H-12 to δ_C_ 153.8 (C-13) and from H-19 to δ_C_ 137.2 (C-16). Both sides of the linear fragment were connected to dibromospirocyclohexadiene moieties through amide bonds according to ^1^H,^13^C-HMBC correlations from H-7 (δ 3.21, 3.63) and 9-NH to C-9 (δ 159.1) as well as from H-7´ (δ 3.19, 3.62) and 9´-NH to C-9´ (δ 159.0). The structure of **1** is closely related to 11-deoxyfistularin-3 (**8**) which was originally isolated from the Caribbean sponge *Aplysina fistularis insularis* [22]. The only difference between **1** and **8** is the lack of one bromine atom in the central benzene ring of compound **1** at C-14. Therefore, compound **1** was named 14-debromo-11-deoxyfistularin-3. The ^13^C chemical shifts assignment of **1** according to the 1,1-ADEQUATE suggested a revision of the chemical shifts of C-2, C-2´, C-6, and C-6´ of the two related compounds 11-deoxyfistularin-3 (**8**) and 14-debromoaraplysilin I (**4**) [18] (Table 1 and Table 2, respectively).

**Table 1:** NMR data (600 MHz, DMSO-*d*_6_) of 14-debromo-11-deoxyfistularin-3 (**1**) and 11-deoxyfistularin-3 (**8**).

position	**1**				**8**			**8**^a^
				
	δ_C_	δ_H_	1,1-ADEQ		δ_C_	δ_H_		δ_C_

1, 1´	73.6, CH; 73.5, CH	3.92, 2H s	2, 2´, 6, 6´		74.1; 74.0	3.93, d (7.9)		74.67; 74.60
2, 2´	113.6, 2C	–	–		113.6	–		121.66^b^
3, 3´	147.1, 2C	–	–		147.6	–		147.92
4, 4´	120.9, C; 120.8, C	–	–		121.4; 121.3	–		115.16^b^
5, 5´	131.2, CH; 131.1, CH	6.57, 2H s	6, 6´		131.7; 131.6	6.57, s; 6.59, s		132.31; 132.15
6, 6´	90.3, C; 90.2, C	–	–		90.8; 90.7	–		91.78; 91.72
7	40.0^c^, CH_2_	3.21, d (18.2)	6, 8		39.9	3.22, d (18.2)		40.27
		3.63, d (18.2)				3.63, d (18.2)		
7´	39.9^c^, CH_2_	3.19, d (18.2) 3.62, d (18.2)	6´, 8´		39.6	3.18, d (18.1) 3.62, d (18.1)		
8, 8´	154.5, C; 154.6, C	–	–		155.0; 154.9	–		155.23; 155.10
9, 9´	159.1, C; 159.0, C	–	–		159.5; 159.4	–		160.44; 160.05
10	36.1, CH_2_	3.36^c^, 2H, overlapped	11		36.7	–		37.13
11	28.6, CH_2_	1.95, 2H qui (6.4)	10, 12		29.9	2.01, qui (7.2)		30.37
12	66.5, CH_2_	4.06, 2H t (6.2)	11		71.7	3.98, t (6.4)		71.51
13	153.8, C	–	–		151.8	–		152.27
14	113.3, CH	7.05, d (8.6)	13, 15		117.8	–		118.35
15	126.7, CH	7.28, dd (1.5, 8.6)	–		130.9	7.58, s		130.90
16	137.2, C	–	–		143.1	–-		143.35
17	130.5, CH	7.52, d (1.5)	–		130.9	7.58, s		130.90
18	110.8, C	–	–		117.8	–		118.35
19	69.9, CH	4.65, dt (4.5, 7.2)	16, 20		69.9	4.69, q (5.3)		70.70
20	46.8, CH_2_	3.35*^c^*, 2H overlapped	–		46.8	3.29, m 3.34^c^		47.99
3, 3´-OMe	59.7, 2CH_3_	3.64, 6H s	–		60.1	3.66, s		59.75
9-NH	–	8.60, t (5.8)	–		–	8.57, t (5.7)		–
9´-NH	–	8.35, t (5.8)	–		–	8.39, t (5.7)		–
1-OH	–	6.36^d^	–		–	6.36, d (7.9)		–
1´-OH	–	6.37^d^	–		–	6.37, d (7.9)		–
19-OH	–	5.54, d (4.5)	–		–	5.73, d (5.3)		–

^a^The ^13^C NMR data were obtained in pyridine-*d*_5_ at 67.5 MHz [22]. ^b^Assignments should be reversed. ^c^Signal obscured by the H_2_O residual signal in DMSO-*d*_6_; chemical shift was obtained from 2D NMR spectra. ^d^Chemical shifts were obtained from the sample before purification. Peaks were not observed in the purified sample.

**Table 2:** ^13^C NMR data (600 MHz, DMSO-*d*_6_) of the central benzene ring of the isolated compounds **1** and **4**.

position	**1**	**4**	**4**^a^

13	153.8	153.3	153.4
14	113.3	111.3	134.4^b^
15	126.7	133.8	133.4
16	137.2	133.4	112.2^b^
17	130.5	129.6	128.8
18	110.8	113.6	113.3

^a^The ^13^C NMR data were obtained in CDCl_3_ [18]. ^b^Assignments should be reversed.

**Figure 4:**
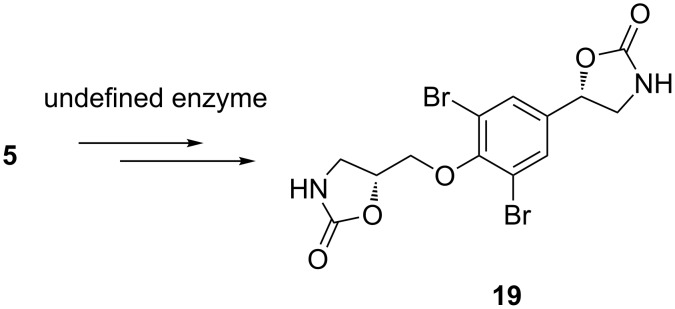
The tissue damage induced chemical conversion from fistularin-3 (**5**) to **19** by an undefined enzyme in the sponge *Aplysina* spp. [36].

The relative configuration of the spiroisoxazoline rings of **1** was investigated using a NOESY experiment. NOEs were observed between δ_H_ 6.36 (1-OH) and 3.60 (H-7); 6.37 (1´-OH) and 3.63 (H-7´); 3.57 (2H, H-5-and H-5´) and 3.21 (2H, H-7 and H-7´) indicated a *trans*-hydroxyspiroisoxazoline ring which was supported by a W-coupling between the olefinic proton H-5 and the methine proton H-1 (^4^*J* ~ 0.7 Hz) [28,33]. An NOE was also observed between δ_H_ 5.54 (19-OH) and δ_H_ 6.37 (1´-OH) suggested that both hydroxy groups are on the same side of the molecule. The absolute configuration of compound **1** was investigated by CD spectroscopy. The CD spectrum of **1** showed positive Cotton effects (λ_max_ 248, Δε +5.16, λ_max_ 285, Δε +4.58) with the same sign and magnitude as observed for (+)-aerothionin (**11**) [25,34–35]. Thus, the absolute configuration of spiroisoxazoline moieties were assigned as 1,1´-(*R*),6,6´-(*S*) (Figure 1). The absolute configuration of C-19 was assigned as 19-(*R*) according to NOE data which is in agreement with the proposed configuration by Molinski and co-workers [34,36]. The configuration of C-19 of fistularin-3 (**5**) was proposed to be the same as of **19**, chemical fragmentation of **5** releasing from the sponge *Aplysina* spp. after induction by tissue damage (Figure 4). However, the conversion from **5** to **19** has not been confirmed. A single data set in the ^13^C NMR spectrum supported the presence of one diastereomer of **1**.

**Figure 5:**
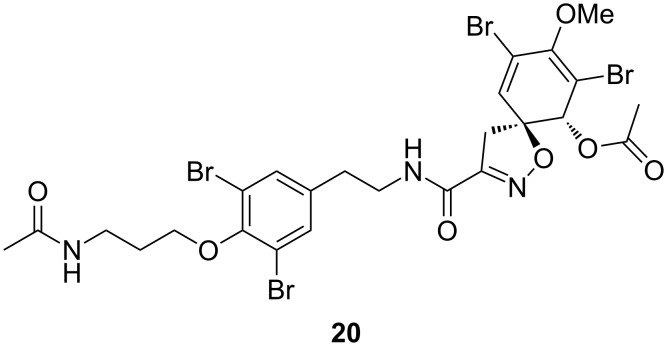
Diacetylhexadellin B (**20**) isolated from sponge *Hexadella* sp.

Compound **2** was isolated as a white solid. MS–ESI(+) data of **2** showed a pseudomolecular ion cluster at *m*/*z* 793/795/797/799/801 [M + Na]^+^ (1:4:6:4:1) indicating a tetrabrominated compound. HRMS–ESI(+) of **2** at *m/z* 793.8320 [M + Na]^+^ suggested a molecular formula of C_23_H_25_Br_4_N_3_O_7_ (calcd for C_23_H_25_^79^Br_4_N_3_O_7_Na, 793.8324). The ^1^H and ^13^C NMR spectra of **2** resemble to **1** except that the spectrum of **2** showed one extra methyl group (δ_H_ 1.80 s; δ_C_ 22.6) and one aromatic proton less (C-14, δ_H_ 7.05 for **1**). According to one set of δ_H_ 3.17 (H-7), 3.61 (H-7), 3.93 (H-1), 6.56 (H-5), δ_C_ 39.7 (C-7), 73.5 (C-1), 90.3 (C-6), 113.1 (C-2), 120.9 (C-4), 131.1 (C-5), 147.1 (C-3), 154.3 (C-8), and 159.0 (C-9) together with ^1^H,^13^C-HMBC correlations revealed that **2** consist of only one spirocyclohexadienylisoxazoline moiety in comparison with **1**. The structure determination of **2** was accomplished based on ^1^H,^1^H-COSY and ^1^H,^13^C-HMBC correlations in the same manner as for **1**. Once again, ^1^H,^1^H-COSY revealed propanamine [H-18 (δ 3.96, 2H)/H-19 (δ 1.91, 2H)/H-20 (δ 3.25)/20-NH (δ 7.86)] and hydroxyethylamine [9-NH (δ 8.39)/H-10 (δ 3.33, 2H)/H-11 (δ 4.67)/11-OH (δ 5.72)] substructures. The signals at δ_H_ 7.57 (2H, s, H-13,17), δ_C_ 117.3 (C-14, 16), 130.4 (C-13, 17), 142.5 (C-12), and 151.4 (C-15) and ^1^H,^13^C-HMBC correlations among those signals showed a 1,2,4,6-dibromophenyl moiety. The substructures were assembled by ^1^H,^13^C-HMBC correlations from H-10 to C-9 and C-12, from H-11 to C-12 and C-13 as well as from δ_H_ H-18 to C-15. The terminal of side chain was connected to an acetamide moiety according to ^1^H,^13^C-HMBC correlations from H-20, 20-NH, and δ_H_ 1.80 (3H; H-22) to δ_C_ 169.1 (C-21). Compound **2** was named aplysinin A. The structure of **2** is similar to right-side portions of **1** and **8** (start at C-10). However, compound **2** contained an acetamide in the left-side portion instead of a ring system in comparison with **8** and showed great similarity with hexadellin B (**10**) isolating from the same organism. Hexadellin B (**10**) was originally isolated from the sponge *Hexadella* sp. The spectroscopic data of **10** was coincidently obtained from diacetylhexadellin B (**20,** Figure 5 and Table 3) [23] which supported the assignment of the acetamide moiety. The relative configuration of the spiroisoxazoline moiety (C-1 and C-6) was determined by comparison of the ^1^H and ^13^C NMR data with **10** and **20**. The NOESY spectrum showed correlations between δ_H_ 6.36 (1-OH) and 3.61 (C-7) as well as between 6.56 (H-6) and 3.17 (H-7) suggesting a *trans*-hydroxyspiroisoxazoline ring similar to compound **1**. An NOE was also observed between δ_H_ 5.72 (11-OH) and 1-OH indicating the same planar alignment of both hydroxy groups. The absolute configuration of spiroisoxazoline moiety was confirmed as 1-(*R*),6-(*S*) by positive Cotton effects (λ_max_ 252, ∆ε +4.77, λ_max_ 283, ∆ε +3.34) comparing to (+)-aerothionin (**11**) in the same manner of **1** [25,37]. The arrangement of 1-OH and 19-OH on the same side of the structure allowed assigning the configuration of C-19. The presence of one diastereomer of **2** was confirmed by a single data set in the ^13^C NMR spectrum.

**Table 3:** NMR data (600 MHz, DMSO-*d*_6_) of aplysinin A (**2**), hexadellin B (**10**), and diacetylhexadellin B (**20**).

position	**2**			**10**			**20**^a^
				
	δ_C_	δ_H_		δ_C_	δ_H_		δ_C_

1	73.5, CH	3.93, d (8.2)		74.0	3.92, d (7.3)		73.1
2	113.1, C	–		113.5	–		122.1^b^
3	147.1, C	–		147.6	–		149.7
4	120.9, C	–		121.4	–		107.8^b^
5	131.1, CH	6.56, s		131.6	6.58, d (0.8)		130.2
6	90.3, C	–		90.7	–		89.9
7	39.7, CH_2_	3.17, d (18.4);		39.6	3.19, d (18.1);		39.9
		3.61, d (18.4)			3.60, d (18.1)		
8	154.3, C	–		154.9	–		153.5
9	159.0, C	–		159.4	–		158.6
10	46.3, CH_2_	3.33, 2H, overlapped		40.4	3.38, m		40.4
11	69.3, CH	4.67, t (6.1)		33.6	2.77, t (7.1)		34.4
12	142.5, C	–		139.5	–		137.2
13,17	130.4, 2CH	7.57, 2H, s		133.5	7.54, s		132.8
14,16	117.3, 2C	–		117.7	–		118.2
15	151.4, C	–		150.9	–		151.5
18	71.3, CH_2_	3.96, 2H, t (6.2)		70.8	4.00, t (6.1)		72.1
19	29.8, CH_2_	1.91, 2H, q (6.9)		28.2	2.08, m		29.4
20	35.7, CH_2_	3.25, 2H, q (6.7)		37.0	3.08, br s		37.7
21	169.1, C	–		–	–		170.0
22	22.6, CH_3_	1.80, 3H, s		–	–		23.6
3-OMe	59.6, CH_3_	3.65, 3H, s		60.1	3.65, s		60.3
9-NH	–	8.39, t (5.9)		–	8.59, t (5.9)		–
1-OH	–	6.36, d (8.2)		–	6.37, d (7.8)		–
11-OH	–	5.72, d (4.4)		–	–		–
20-NH	–	7.86, t (5.3)		–	–		–

^a^The ^13^C NMR data were obtained in CDCl_3_ [23]. ^b^Assignments should be reversed.

**Figure 6:**
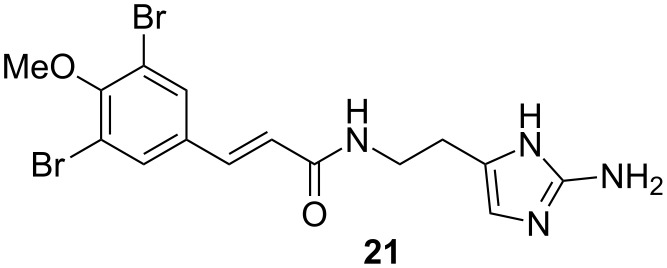
Bromotyrosine alkaloid (**21**) isolated from the sponge *Verongula* sp.

Compound **3** was isolated together with *N*-methylaerophobin-2 (**15**) as a mixture (approximate ratio 1:5). The ESIMS spectrum exhibited a 1:2:1 ion cluster at *m/z* 457/459/461, indicating the presence of two bromine atoms. The HRMS–ESI(+) spectrum revealed a pseudomolecular ion [M + H]^+^ at *m/z* 456.9892, which indicated a molecular formula of C_16_H_18_Br_2_N_4_O_2_ (calcd for C_16_H_19_^79^Br_2_N_4_O_2_, 456.9875), containing nine DBEs. Two singlet aromatic protons δ_H_ 7.88 (δ_C_ 131.7) suggested a tetrasubstituted benzene pattern which was confirmed by ^1^H,^13^C-HMBC correlations; from δ_H_ 7.88 (2H, H-2, H-6) to δ_C_ 118.5 (C-3, C-5), δ_C_ 154.4 (C-4), δ_C_ 131.7 (C-2, C-6), and δ_C_ 135.7 (C-7), from δ_H_ 3.82 (4-OMe) to C-4. The connection of the benzene fragment to the *E*-vinyl moiety was confirmed by HMBC correlations from two olefinic protons δ_H_ 7.33 (d, *J* = 15.8 Hz, H-7) and δ_H_ 6.66 (d, *J* = 15.8 Hz, H-8) to δ_C_ 134.9 (C-1). ^1^H,^1^H-COSY correlations in between 9-NH (δ 8.17)/H-10 (δ 3.20, 2H)/H-11 (δ 1.71, 2H)/H-12 (δ 2.45, 2H) indicated a propanamine fragment which was connected to a 2-aminoimidazole moiety according to ^1^H,^13^C-HMBC correlations from H-12 to δ_C_ 126.8 (C-13) and 109.2 (C-14) (Table 4). The two substructures are connected through an amide bond according to the ^1^H,^13^C-HMBC correlations from H-7, 9-NH, and H-10 to δ_C_ 165.0 (C-9). The structure of **3** is very similar to compound **21** (Figure 6) which was isolated from the Caribbean sponge *Verongula* sp. [38]. In comparison with **21**, compound **3** showed one extra aliphatic carbon and was named aplysinin B.

**Table 4:** NMR data of aplysinin B (**3**) (600 MHz, DMSO-*d*_6_) and compound **21**.

position	**3**			**21**^a^
		
	δ_C_	δ_H_		δ_C_

1	134.9, C	–		136.36
2, 6	131.7, CH	7.88, 2H, s		132.98
3, 5	118.5, C	–		119.48
4	154.4, C	–		154.46
7	135.7, CH	7.33, d (15.8)		138.42
8	124.9, CH	6.66, d (15.8)		123.95
9	165.0, C	–		167.50
10	38.4, CH_2_	3.20, 2H, m		39.27
11	28.1, CH_2_	1.71, 2H, m		25.92
12	22.0, CH_2_	2.45, 2H, m		125.89
13	126.8, C	–		110.87
14	109.2, CH	6.60, s		146.43
15	147.4, C	–		–
4-OMe	61.0, CH_3_	3.82, s		61.26
9-NH	–	8.17, t (5.58)		–

^a^The ^13^C NMR data were obtained in CD_3_OD [38].

The new compounds 14-debromo-11-deoxyfistularin-3 (**1**) and aplysinin A (**2**) were tested for their antimicrobial activity against different Gram-positive and Gram-negative bacteria, fungi, and for their antiproliferative activity. Aplysinin B (**3**) was not subjected to any biological activity test due to the minute amount and its existence as the minor compound of a mixture. The results showed that **1** and **2** exhibited mild cytotoxic activity against KB-31 epidermoid carcinoma cells (IC_50_ = 69 and 26 µM, respectively). Only **2** showed mild toxicity against the breast cancer cell line MCF-7 and to FS4-LTM conditional immortalization human fibroblasts (IC_50_ = 78 and 32 µM, respectively). The cytotoxicities of the known compounds (**4**–**17**) are also listed in Table 5. None of the isolated compounds showed any antimicrobial activity.

**Table 5:** Cytotoxicity of the isolated compounds (IC_50_).^a^

compound	IC_50_ [µM]			
	L929	KB-31	MCF-7	FS4-LTM

**1**	–	68.8	–	–
**2**	–	25.8	77.5	32.2
**4**	94.3	–	78.6	–
**5**	–	–	206.9	–
**6**	117.6	88.2	–	–
**7**	–	–	60.0	–
**8**	–	–	47.2	87.3
**10**	–	–	90.6	73.4
**15**	55.9	48.9	–	–
**16**	–	–	96.3	–
**17**	–	–	64.8	–

^a^Compounds with no activity are not listed in the table.

## Experimental

UV spectra were recorded during HPLC separation with a DAD detector (JASCO MD-2010 Plus). CD spectra were recorded on a JASCO J-810 spectropolarimeter. Low and high resolution ESIMS was performed with a Bruker micrOTOF*_LC_* mass spectrometer. Mass calibration was performed using sodium formate cluster ions prior each measurement. ^1^H and ^13^C spectra were recorded on a Bruker Avance 600 NMR spectrometer equipped with a cryo platform (^1^H at 600 MHz, ^13^C at 150 MHz) and a Bruker Avance NMR spectrometer (^1^H at 400 MHz, ^13^C at 100 MHz). All NMR experiments were measured at a temperature of 303 K using DMSO-*d*_6_ (δ_H_ 2.50, δ_C_ 39.5) as internal standard. HPLC separation was achieved by Jasco PU-1580 using a Kromasil RP18 column (16 mm × 250 mm, 5 µm) and a Kromasil RP18 column (1 mm × 50 mm, 5 µm) and was eluted with gradient H_2_O (0.1% TFA) and MeCN (0.1% TFA).

The sponge *Aplysina lacunosa* was collected by SCUBA diving at a depth of 8 m from Stirrup Cay in the Bahamas in June 2008. The sample was immediately frozen and kept at −20 °C until extraction. A voucher specimen of this species is deposited in AG Köck, Alfred-Wegener-Institut, Helmholtz-Zentrum für Polar- und Meeresforschung (voucher number: *Aplysina lacunosa* 08/21). The freeze-dried sponge (200 g) was extracted three times with CH_2_Cl_2_/MeOH (1:1, v/v) at room temperature. The filtrates were pooled and evaporated to yield 24.3 g of crude extract which was further partitioned between *n*-hexane and MeOH. The MeOH extract was then partitioned between EtOAc and H_2_O. The EtOAc fraction was further purified by vacuum liquid chromatography using silica gel eluting with stepwise gradient from 100:0 to 80:20 (CH_2_Cl_2_/MeOH, v/v). The fraction eluted with 97:3 (CH_2_Cl_2_/MeOH, v/v) was concentrated and further purified by HPLC using an RP C18 column (stepwise gradient 60:40, 40:60, and 20:80 H_2_O/MeCN, v/v) to yield **5** (198.0 mg), **11** (263.0 mg), **12** (445.0 mg), **13** (55.0 mg), **14** (7.9 mg), **16** (15.0 mg), **17** (9.1 mg), and two other fractions. The first fraction was purified using reversed-phase HPLC [52:48 H_2_O (0.1% TFA)/MeCN (0.1% TFA), v/v] to obtain **7** (3.2 mg), **8** (4.5 mg), **9** (9.6 mg) and following with analytical RP18 column [gradient 60:40 to 20:80 H_2_O (0.1% TFA)/MeCN (0.1% TFA), v/v] yielding **1** (0.8 mg). The other fraction was purified using RP18 HPLC [60:40 H_2_O (0.1% TFA)/MeCN (0.1% TFA), v/v] and follow with an analytical RP18 column [65:35 H_2_O (0.1% TFA)/MeCN (0.1% TFA), v/v] yielding **2** (0.8 mg) and **18** (5.3 mg). The 94:6 (CH_2_Cl_2_/MeOH, v/v) fraction was purified by HPLC using a RP C18 column (gradient 80:20 to 40:60 H_2_O/MeCN, v/v) yielding **6** (10.7 mg), **10** (25.5 mg), **15** (5.0 mg), and one fraction which was purified using reversed-phase HPLC [70:30 H_2_O (0.1% TFA)/MeCN (0.1% TFA), v/v] to get a mixture of **3** and **15** (3.5 mg) as well as **4** (3.0 mg).

### Biological activity test

The antimicrobial activities of isolated compounds were evaluated against five microorganisms [Gram-positive: *Straptococcus aureus* (MRSA and MSSA) and *Micrococcus luteus*; Gram-negative: *Peumonia aruginosa* and *Klebsiella pneumonia*] and antifungal *Candida albicans* using microdilution technique. The MIC was defined as lowest concentration that shows 50% growth inhibition after 24 hour incubation.

### Cytotoxicity assay

The cytotoxicity was determined using WST-1 cell proliferation assays. Targeting cell lines are L929 mouse fibroblasts, KB-31 epidermoid carcinoma, and MCF-7 breast cancer cell lines which were incubated for 5 days with the test substances. The acute toxicity was determined using the FS4-LTM conditional immortalization human fibroblasts cell line which was incubated for 24 hours with the test compounds.

### Experimental data

14-Debromo-11-deoxyfistularin-3 (**1**): white solid; UV (DAD) λ_max_ 226 nm; CD (MeOH) λ_max_ 248 nm (∆ε +5.16), 288 nm (∆ε +4.55); ^1^H NMR and ^13^C NMR see Table 1; HRMS–ESI(+) *m*/*z* = 1036.7844 [M + Na]^+^ (calcd for C_31_H_31_^79^Br_5_N_4_O_10_Na, 1036.7855, Δ*m* = 1.1 ppm).

Aplysinin A (**2**): white solid; UV (DAD) λ_max_ 225 nm; CD (MeOH) λ_max_ 252 nm (∆ε +4.77), 283 nm (∆ε +3.34); ^1^H NMR and ^13^C NMR see Table 3; HRMS–ESI(+) *m*/*z* = 793.8320 [M + Na]^+^ (calcd for C_23_H_25_^79^Br_4_N_3_O_7_Na, 793.8324, Δ*m* = 0.4 ppm).

Aplysinin B (**3**): white solid; UV (DAD) λ_max_ 227 nm; ^1^H NMR and ^13^C NMR see Table 4; HRMS–ESI(+) *m*/*z* = 456.9892 [M + H]^+^ (calcd for C_16_H_19_^79^Br_2_N_4_O_2_, 456.9875, Δ*m* = 1.7 ppm).

## Supporting Information

File 1 1D, 2D NMR, and CD spectra of three new compounds. 1D NMR, mass and CD spetra of all known isolated compounds.

## References

[1] Sharma G M, Burkholder P R (1967). Tetrahedron Lett.

[2] Gunasekera S P, Cross S S (1992). J Nat Prod.

[3] Kobayashi J, Tsuda M, Agemi K, Shigemori H, Ishibashi M, Sasaki T, Mikami Y (1991). Tetrahedron.

[4] Andersen R J, Faulkner D J (1973). Tetrahedron Lett.

[5] Gao H, Kelly M, Hamann M T (1999). Tetrahedron.

[6] Okamoto Y, Ojika M, Kato S, Sakagami Y (2000). Tetrahedron.

[7] Tsuda M, Shigemori H, Ishibashi M, Kobayashi J (1992). Tetrahedron Lett.

[8] Yagi H, Matsunaga S, Fusetani N (1993). Tetrahedron.

[9] Ichiba T, Scheuer P J, Kelly-Borges M (1993). J Org Chem.

[10] Ross S A, Weete J D, Schinazi R F, Wirtz S S, Tharnish P, Scheuer P J, Hamann M T (2000). J Nat Prod.

[11] Tsukamoto S, Kato H, Hirota H, Fusetani N (1996). Tetrahedron.

[12] Mierzwa R, King A, Conover M A, Tozzi S, Puar M S, Patel M, Coval S J, Pomponi S A (1994). J Nat Prod.

[13] Acosta A L, Rodriguez A D (1992). J Nat Prod.

[14] Tabudravu J N, Jaspars M (2002). J Nat Prod.

[15] Yang X, Davis R A, Buchanan M S, Duffy S, Avery V M, Camp D, Quinn R J (2010). J Nat Prod.

[16] Galeano E, Martínez A, Thomas O P, Robledo S, Munoz D (2012). Quim Nova.

[17] Galeano E, Thomas O P, Robledo S, Munoz D, Martinez A (2011). Mar Drugs.

[18] James D M, Kunze H B, Faulkner D J (1991). J Nat Prod.

[19] Gopichand Y, Schmitz F J (1979). Tetrahedron Lett.

[20] Kernan M R, Cambie R C, Bérgquist P R (1990). J Nat Prod.

[21] Mancini I, Guella G, Laboute P, Debitus C, Pietra F (1993). J Chem Soc, Perkin Trans 1.

[22] Compagnone R S, Avila R, Suárez A I, Abrams O V, Rangel H R, Arvelo F, Piña I C, Merentes E (1999). J Nat Prod.

[23] Morris S A, Andersen R J (1989). Can J Chem.

[24] Fattorusso E, Minale L, Sodano G, Moody K, Thomson R H (1970). J Chem Soc D.

[25] McMillan J A, Paul I C, Goo Y M, Rinehart K L, Krueger W C, Pschigoda L M (1981). Tetrahedron Lett.

[26] Ciminiello P, Costantino V, Fattorusso E, Magno S, Mangoni A (1994). J Nat Prod.

[27] Assmann M, Wray V, van Soest R W M, Proksch P (1998). Z Naturforsch, C.

[28] Minale L, Sodano G, Chan W R, Chen A M (1972). J Chem Soc, Chem Commun.

[29] Abou-Shoer M I, Shaala L A, Youssef D T A, Badr J M, Habib A-A M (2008). J Nat Prod.

[30] Nishiyama S, Yamamura S (1985). Bull Chem Soc Jpn.

[31] Tilvi S, Rodrigues C, Naik C G, Parameswaran P S, Wahidhulla S (2004). Tetrahedron.

[32] Köck M, Reif B, Fenical W, Griesinger C (1996). Tetrahedron Lett.

[33] Fulmor W, van Lear G E, Morton G O, Mills R D (1970). Tetrahedron Lett.

[34] Rogers E W, de Oliveira M F, Berlinck R G S, König G M, Molinski T F (2005). J Nat Prod.

[35] Rogers E W, Molinski T F (2007). J Nat Prod.

[36] Ebel R, Brenzinger M, Kunze A, Gross H J, Proksch P (1997). J Chem Ecol.

[37] Ciminiello P, Dell'Aversano C, Fattorusso E, Magno S, Pansini M (1999). J Nat Prod.

[38] Ciminiello P, Fattorusso E, Magno S, Pansini M (1994). J Nat Prod.

