# Supplementary material for: Bromotyrosine-derived alkaloids from the Caribbean sponge Aplysina lacunosa
Source: Beilstein J Org Chem. 2015 Nov 26;11:2334–42. doi: 10.3762/bjoc.11.254 (PMC4685874; doi:10.3762/bjoc.11.254)
Supplement: File 1 — 1D, 2D NMR, and CD spectra of three new compounds. 1D NMR, mass and CD spetra of all known isolated compounds. [file Beilstein_J_Org_Chem-11-2334-s001.pdf]

Supporting Information

for

Bromotyrosine-derived alkaloids from the Caribbean sponge *Aplysina lacunosa*

Qun Göthel, Thanchanok Sirirak, and Matthias Köck*

Alfred-Wegener-Institut, Helmholtz-Zentrum für Polar- und Meeresforschung,

Am Handelshafen 12, 27570 Bremerhaven, Germany

Email: Matthias Köck - mkoeck@awi.de

* Corresponding author

1D, 2D NMR, and CD spectra of three new compounds. 1D NMR, mass and CD spectra of all known isolated compounds

Figure S1: 1D ^{1}H-NMR spectrum of 14-debromo-11-deoxyfistularin-3 (**1**) S4

Figure S2: 1D ^{13}C-NMR spectrum of 14-debromo-11-deoxyfistularin-3 (**1**) S4

Table S1: NMR data (^{13}C, ^{1}H, ^{1}H,^{1}H-COSY, ^{1}H,^{1}H-HMBC, 1,1-ADEQUATE, and ^{1}H,^{1}H-NOESY) of 14-debromo-11-deoxyfistularin-3 (**1**) S5

Figure S3: 2D ^{1}H,^{13}C-HSQC spectrum of 14-debromo-11-deoxyfistularin-3 (**1**) S6

Figure S4: 2D ^{1}H,^{13}C-HMBC spectrum of 14-debromo-11-deoxyfistularin-3 (**1**) S6

Figure S5: 2D ^{1}H,^{13}C-HMBC spectrum of 14-debromo-11-deoxyfistularin-3 (**1**) S7

Figure S6: 2D ^{1}H,^{1}H-COSY spectrum of 14-debromo-11-deoxyfistularin-3 (**1**) S7

Figure S7: 2D 1,1-ADEQUATE spectrum of 14-debromo-11-deoxyfistularin-3 (**1**) S8

Figure S8: 2D ^{1}H,^{1}H-NOESY spectrum of 14-debromo-11-deoxyfistularin-3 (**1**) S8

Figure S9: Circular dichroism spectrum of 14-debromo-11-deoxyfistularin-3 (**1**) S9

**Figure S10:**	1D ^{1}H-NMR spectrum of aplysinin A (**2**)	S10
**Figure S11:**	1D ^{13}C-NMR spectrum of aplysinin A (**2**)	S10
**Table S2:**	NMR data (^{13}C, ^{1}H, ^{1}H,^{1}H-COSY, ^{1}H,^{1}H-HMBC, 1,1-ADEQUATE, and ^{1}H,^{1}H-NOESY) of aplysinin A (**2**)	S11
**Figure S12:**	2D ^{1}H,^{13}C-HSQC spectrum of aplysinin A (**2**)	S12
**Figure S13:**	2D ^{1}H,^{13}C-HMBC spectrum of aplysinin A (**2**)	S12
**Figure S14:**	2D ^{1}H,^{13}C-HMBC spectrum of aplysinin A (**2**)	S13
**Figure S15:**	2D ^{1}H,^{1}H-COSY spectrum of aplysinin A (**2**)	S13
**Figure S16:**	2D 1,1-ADEQUATE spectrum of aplysinin A (**2**)	S14
**Figure S17:**	2D ^{1}H,^{1}H-NOESY spectrum of aplysinin A (**2**)	S14
**Figure S18:**	Circular dichroism spectrum of aplysinin A (**2**)	S15
**Figure S19:**	1D ^{1}H-NMR spectrum of aplysinin B (**3**)	S16
**Figure S20:**	1D ^{13}C-NMR spectrum of aplysinin B (**3**)	S16
**Table S3:**	NMR data (^{13}C, ^{1}H, ^{1}H,^{1}H-COSY, and ^{1}H,^{1}H-HMBC) of aplysinin B (**3**)	S17
**Figure S21:**	2D ^{1}H,^{13}C-HSQC spectrum of aplysinin B (**3**)	S17
**Figure S22:**	2D ^{1}H,^{13}C-HMBC spectrum of aplysinin B (**3**)	S18
**Figure S23:**	2D ^{1}H,^{1}H-COSY spectrum of aplysinin B (**3**)	S18
**Figure S24:**	1D ^{1}H-NMR spectrum of 14-debromoaraplysillin I (**4**)	S19
**Figure S25:**	ESI–MS(+) spectral data of 14-debromoaraplysillin I (**4**).	S19
**Figure S26:**	Circular dichroism spectrum of 14-debromoaraplysillin I (**4**)	S20
**Figure S27:**	1D ^{1}H-NMR spectrum of fistularin-3 (**5**)	S20
**Figure S28:**	ESI–MS(+) spectral data of fistularin-3 (**5**)	S21
**Figure S29:**	Circular dichroism spectrum of fistularin-3 (**5**)	S21
**Figure S30:**	1D ^{1}H-NMR spectrum of 11,19-dideoxyfistularin-3 (**6**)	S22
**Figure S31:**	1D ^{1}H-NMR spectrum of 19-deoxyfistularin-3 (**7**)	S22
**Figure S32:**	ESI-MS spectral data of 19-deoxyfistularin-3 (**7**).	S23
**Figure S33:**	Circular dichroism spectrum of 19-deoxyfistularin-3 (**7**)	S23
**Figure S34:**	1D ^{1}H-NMR spectrum of 11-deoxyfistularin-3 (**8**)	S24
**Figure S35:**	ESI-MS(+) spectral data of 11-deoxyfistularin-3 (**8**)	S24
**Figure S36:**	Circular dichroism spectrum of 11-deoxyfistularin-3 (**8**)	S25
**Figure S37:**	1D ^{1}H-NMR spectrum of 11-ketofistularin-3 (**9**)	S25

Figure S38: ESI–MS(+) spectral data of 11-ketofistularin-3 (**9**). S26
Figure S39: Circular dichroism spectrum of 11-ketofistularin-3 (**9**) S26
Figure S40: 1D ^{1}H-NMR spectrum of hexadelin B (**10**) S27
Figure S41: ESI–MS(+) spectral data of hexadelin B (**10**) S27
Figure S42: 1D ^{1}H-NMR spectrum of aerothionin (**11**) S28
Figure S43: ESI–MS(+) spectral data of aerothionin (**11**) S28
Figure S44: Circular dichroism spectrum of aerothionin (**11**) S29
Figure S45: 1D ^{1}H-NMR spectrum of 11-hydroxyaerothionin (**12**) S29
Figure S46: ESI-MS(+) spectral data of 11-hydroxyaerothionin (**12**) S30
Figure S47: Circular dichroism spectrum of 11-hydroxyaerothionin (**12**) S30
Figure S48: 1D ^{1}H-NMR spectrum of 11-oxoaerothionin (**13**) S31
Figure S49: ESI–MS(+) spectral data of 11-oxoaerothionin (**13**) S31
Figure S50: Circular dichroism spectrum of 11-oxoaerothionin (**13**) S32
Figure S51: 1D ^{1}H-NMR spectrum of 11-oxo-12-hydroxyaerothionin (**14**) S32
Figure S52: ESI–MS(+) spectral data of 11-oxo-12-hydroxyaerothionin (**14**) S33
Figure S53: Circular dichroism spectrum of 11-oxo-12-hydroxyaerothionin (**14**) S33
Figure S54: 1D ^{1}H-NMR spectrum of *N*-methylaerophobin-2 (**15**) S34
Figure S55: ESI–MS(+) spectral data of *N*-methylaerophobin-2 (**15**) S34
Figure S56: 1D ^{1}H-NMR spectrum of aeroplysinin-2 (**16**) S35
Figure S57: ESI–MS(+) spectral data of aeroplysinin-2 (**16**) S35
Figure S58: Circular dichroism spectrum of aeroplysinin-2 (**16**) S36
Figure S59: 1D ^{1}H-NMR spectrum of subereaphenol B (**17**) S36
Figure S60: ESI–MS(+) spectral data of subereaphenol B (**17**) S37
Figure S51: 1D ^{1}H-NMR spectrum of unnamed bromytyrosine (**18**) S37
Figure S62: ESI–MS(+) spectral data of unnamed bromytyrosine (**18**) S38
Figure S63: Circular dichroism spectrum of unnamed bromytyrosine (**18**) S38

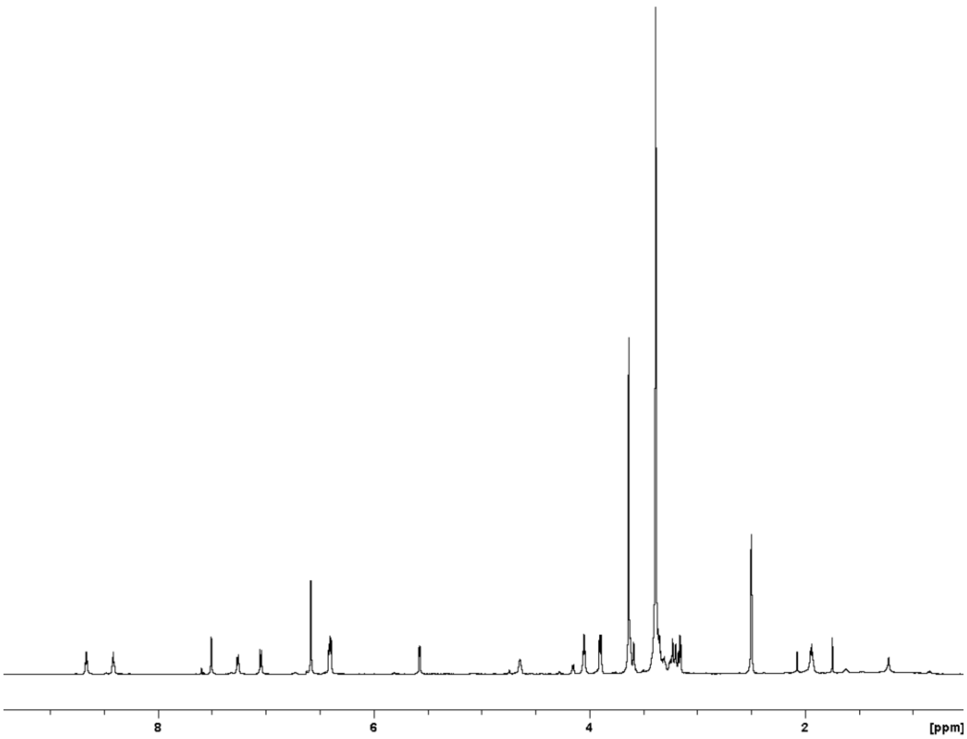

Figure S1: 1D ^{1}H-NMR spectrum of 14-debromo-11-deoxyfistularin-3 (**1**) in DMSO-d_6, 303 K, 600 MHz.

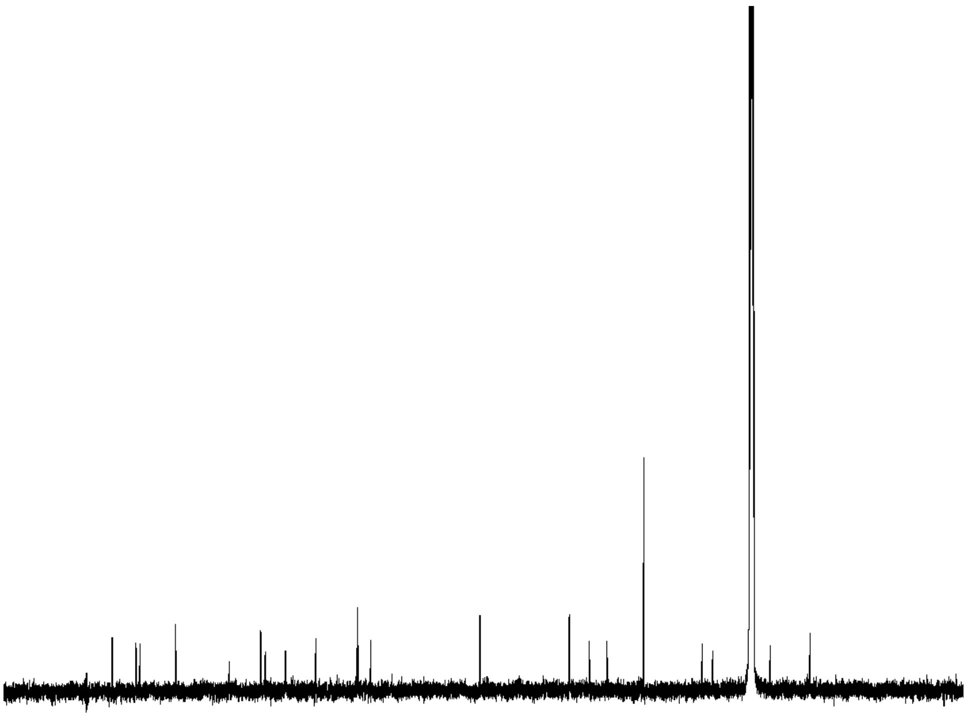

Figure S2: 1D ^{13}C-NMR spectrum of 14-debromo-11-deoxyfistularin-3 (**1**) in DMSO-d_6, 303 K, 150 MHz.

Table S1: NMR data (^{13}C, ^{1}H, ^{1}H,^{1}H-COSY, ^{1}H,^{13}C-HMBC, 1,1-ADEQUATE, and ^{1}H,^{1}H-NOESY) of 14-debromo-11-deoxyfistularin-3 (**1**) in DMSO-d_6

position	δ_C	δ_H	^{1}H,^{1}H-COSY	^{1}H,^{13}C-HMBC	1,1-ADEQ	^{1}H,^{1}H-NOESY
1/1´	73.6<br>73.5	3.92	---	2/2´, 3/3´,<br>5/5´, 6/6´	2/2´,<br>6/6´	---<br>---
2/ 2´	113.6, 2C	---	---	---	---	---
3/ 3´	147.1, 2C	---	---	---	---	---
4/ 4´	120.9,<br>120.8	---	---	---	---	---
5/5´	131.2,<br>131.1	6.57	---	1/1´, 6/6´, 7/7´	6/ 6´	---
6/ 6´	90.3<br>90.2	---	---	---	---	7/ 7´
7/	40.0	3.21<br>3.63	---<br>---	1/1´, 5/5´,<br>6/6´, 8/8´, 9/9´	6/6´, 8/8´	6
7´	39.9	3.19<br>3.62	---			6´
8/ 8´	154.5<br>154.6	---	---	---	---	---
9/ 9´	159.1<br>159.0	---	---	---	---	---
10	36.1	3.36	9-NH, 11	9	11	---
11	28.6	1.95	10, 12	---	10, 12	---
12	66.5	4.06	11	10, 11, 13	11	---
13	153.8	---	---	---	---	---
14	113.3	7.05	15	13, 15	13, 15	---
15	126.7	7.28	14	---	---	---
16	137.2	---	---	---	---	---
17	130.5	7.52	---	---	---	---
18	110.8	---	---	---	---	---
19	69.9	4.65	20	15, 16, 17, 20	16, 20	15, 17,<br>9´-NH
20	46.8	3.35	19, 9´-NH	9´	---	---
3/ 3´-OMe	59.7, 2C	3.64	---	---	---	---
9-NH	---	8.60	10	9, 10	---	---
9´-NH	---	8.35	20	20	---	---
1-OH	---	6.36	---	---	---	7
1´-OH	---	6.37	---	---	---	7´, 19-OH
19-OH	---	5.54	---	---	---	1´-OH

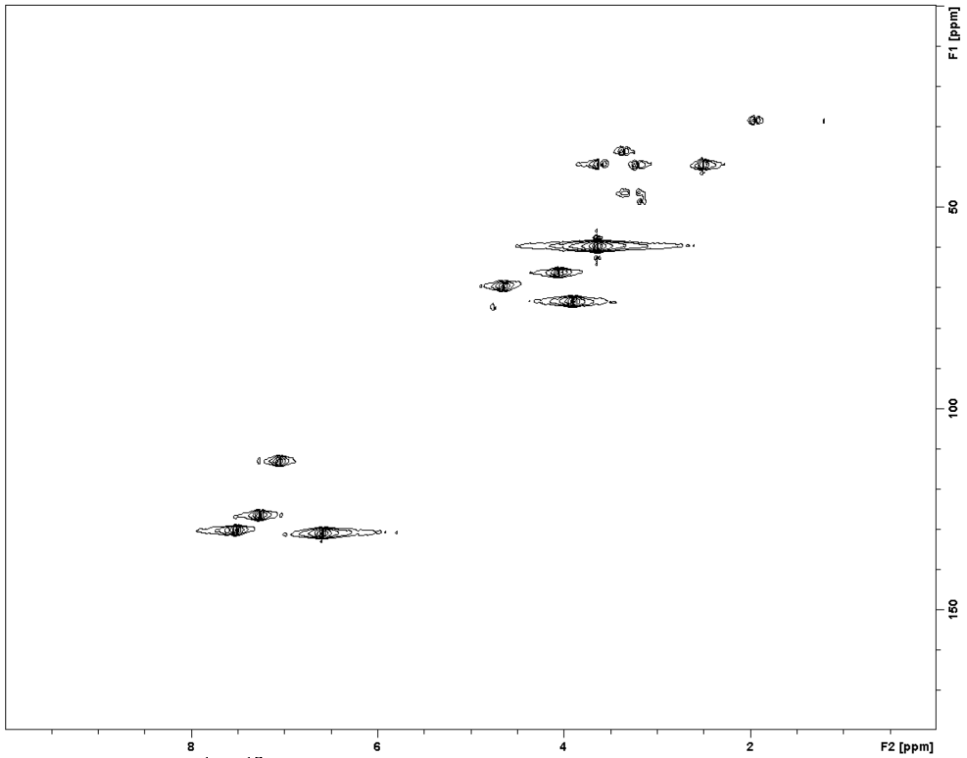

Figure S3: 2D $^{1}H,^{13}C$-HSQC spectrum of 14-debromo-11-deoxyfistularin-3 (**1**) in DMSO-d_6, 303 K.

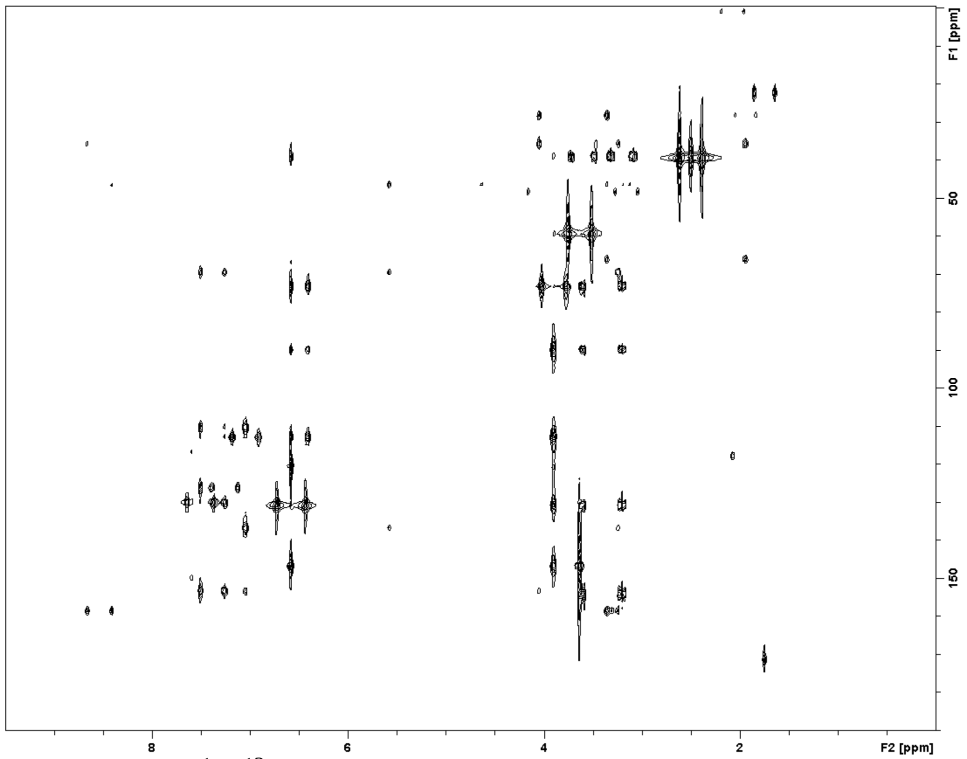

Figure S4: 2D $^{1}H,^{13}C$-HMBC spectrum of 14-debromo-11-deoxyfistularin-3 (**1**) in DMSO-d_6, 303 K.

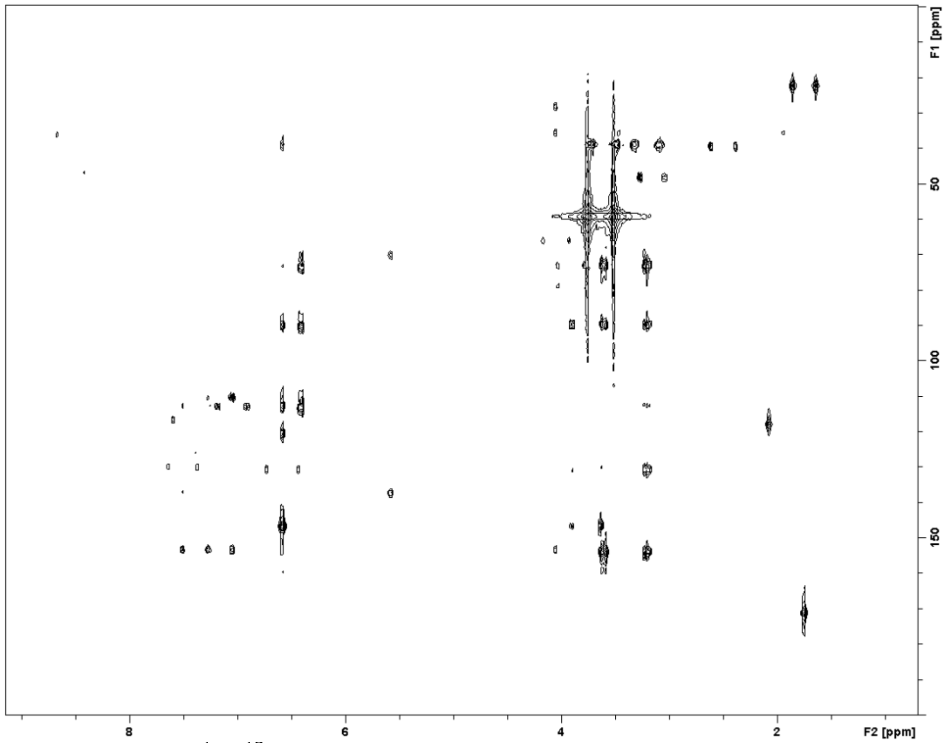

Figure S5: 2D $^{1}H,^{13}C$-HMBC spectrum of 14-debromo-11-deoxyfistularin-3 (**1**) in DMSO-d_6, D6 = 0.2500, 303 K.

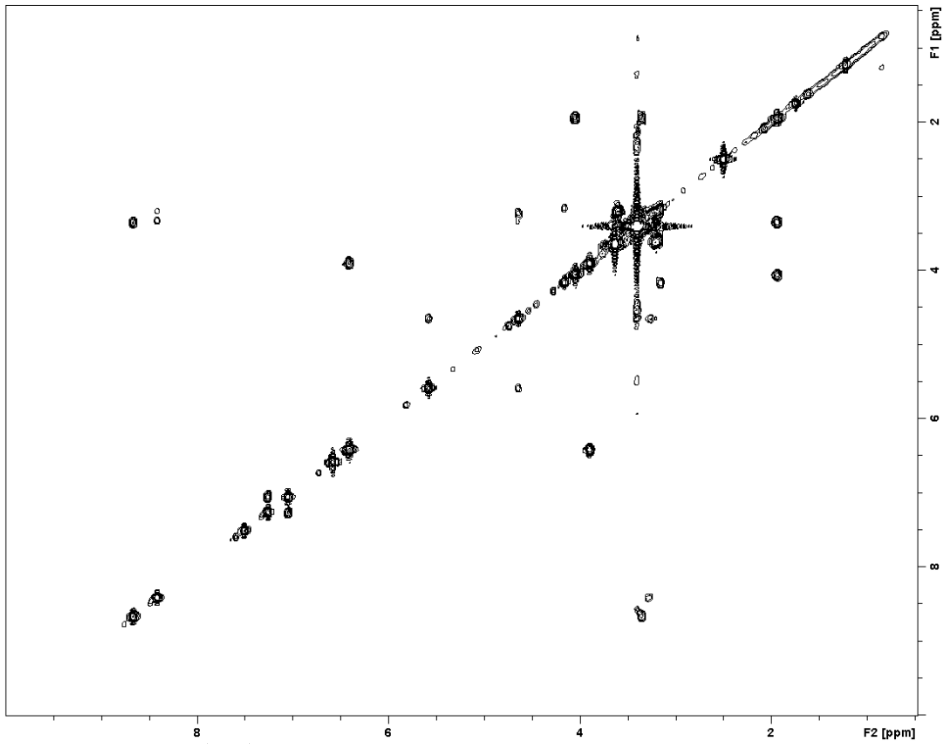

Figure S6: 2D $^{1}H,^{1}H$-COSY spectrum of 14-debromo-11-deoxyfistularin-3 (**1**) in DMSO-d_6, 303 K.

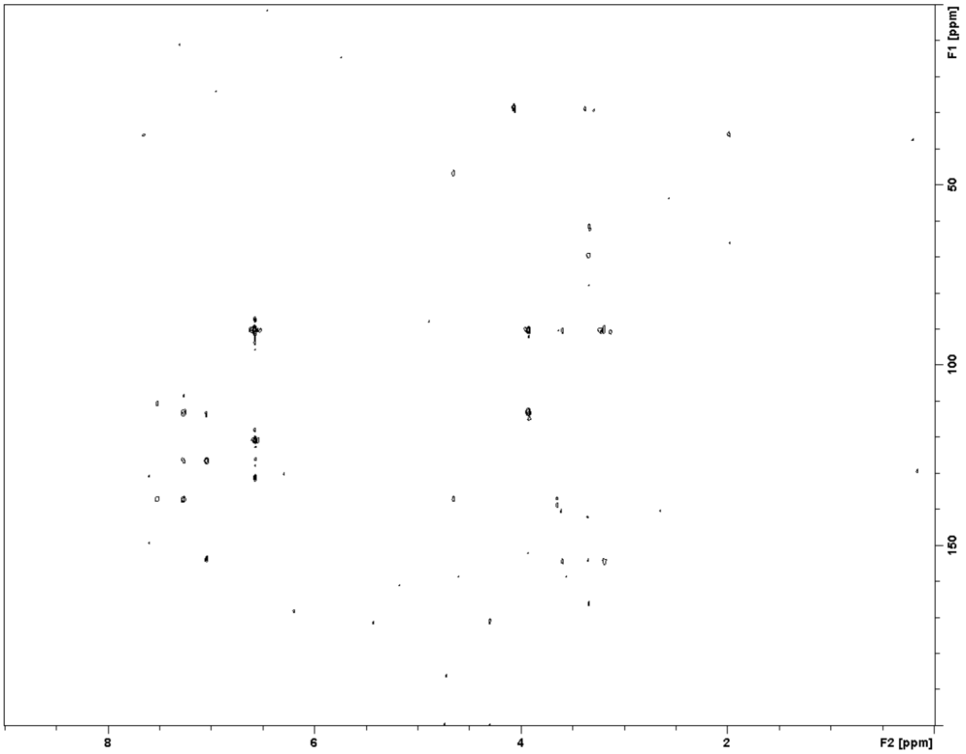

Figure S7: 2D 1,1-ADEQUATE spectrum of 14-debromo-11-deoxyfistularin-3 (**1**) in DMSO-d_6, 303 K.

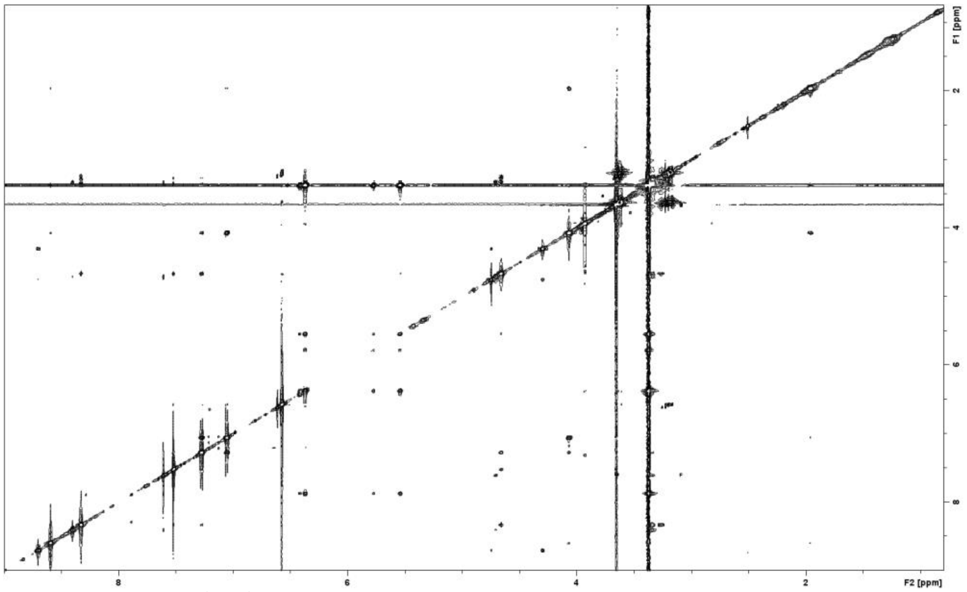

Figure S8: 2D ^{1}H,^{1}H-NOESY spectrum of 14-debromo-11-deoxyfistularin-3 (**1**) in DMSO-d_6, 303 K.

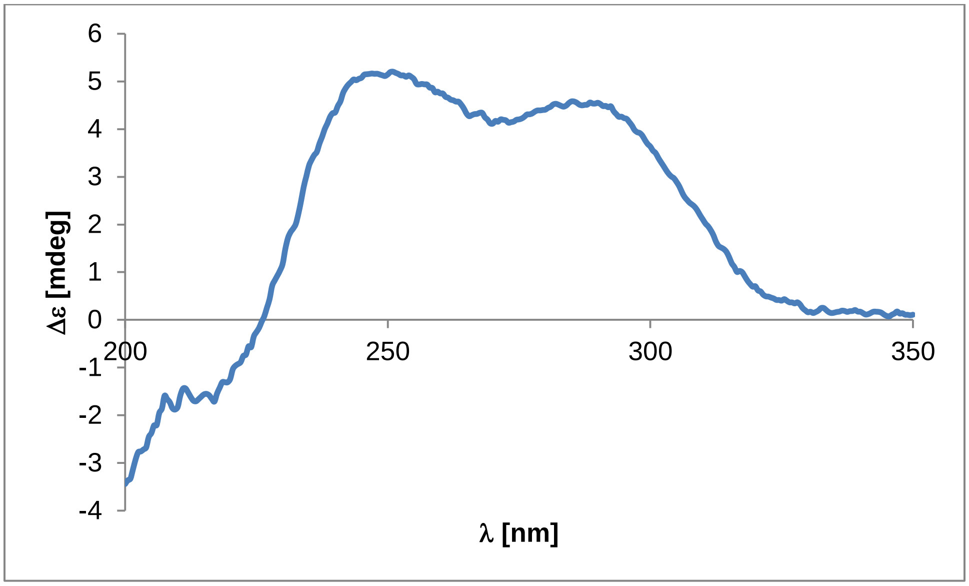

Figure S9: Circular dichroism spectrum of 14-debromo-11-deoxyfistularin-3 (**1**) (c = 2.0 × 10^{-4} M, MeOH, 25 °C).

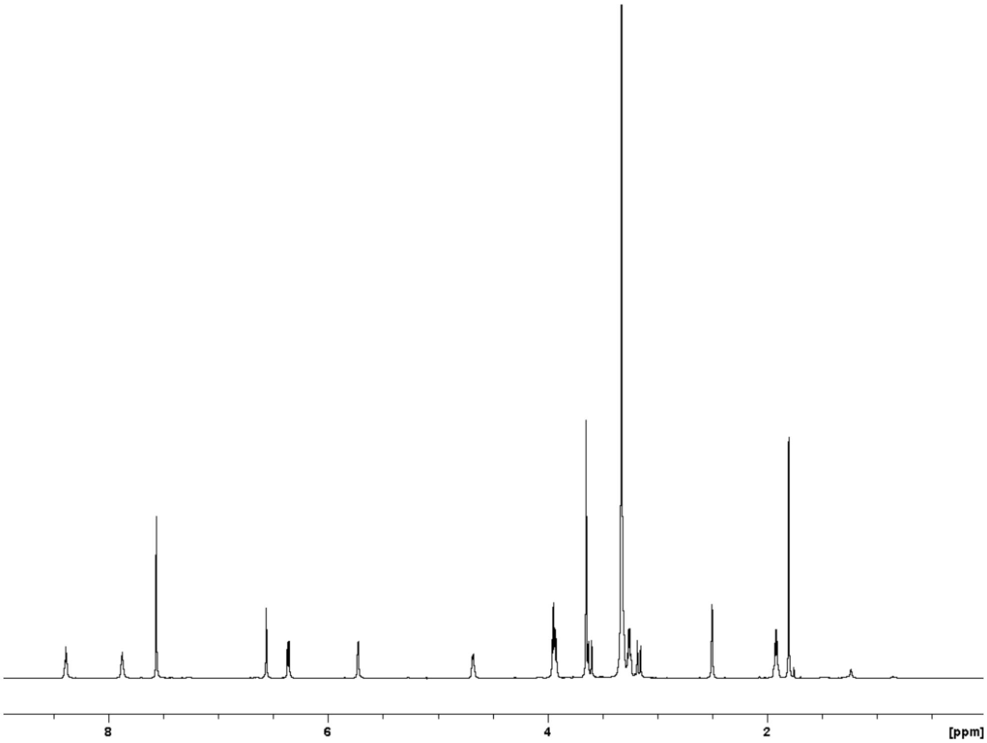

Figure S10: 1D ^{1}H-NMR spectrum of aplysinin A (**2**) in DMSO-d_6, 303 K, 600 MHz.

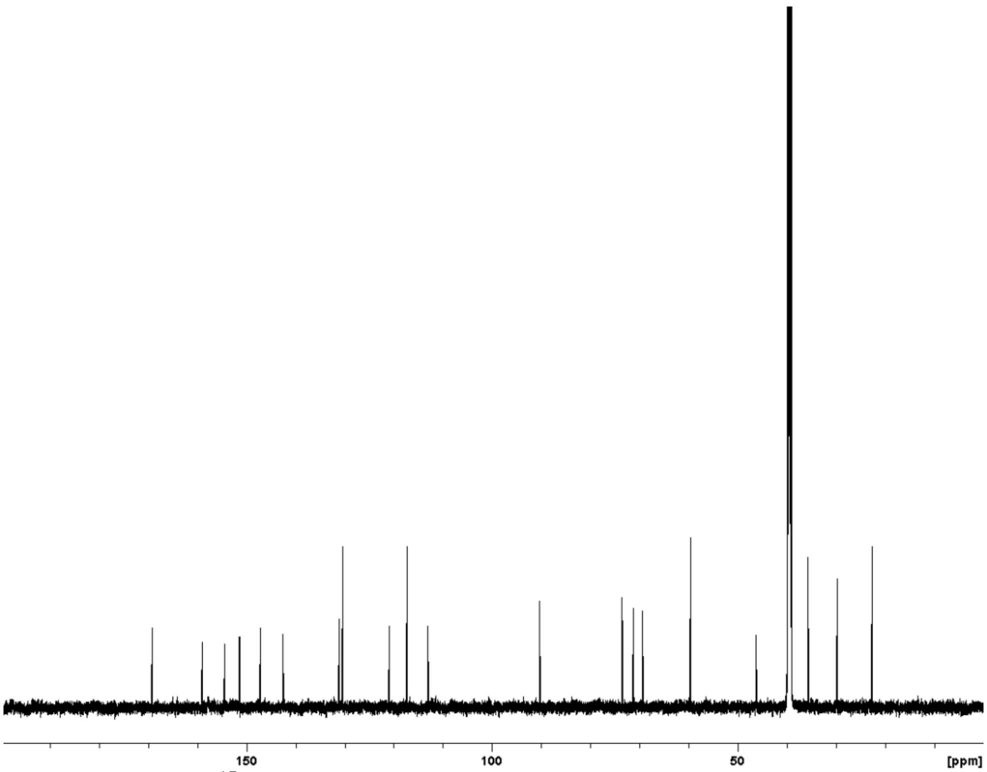

Figure S11: 1D ^{13}C-NMR spectrum of aplysinin A (**2**) in DMSO- d_6, 303 K, 150 MHz.

Table S2: NMR data (^{13}C, ^{1}H, ^{1}H,^{1}H-COSY, ^{1}H,^{13}C-HMBC, 1,1-ADEQUATE, and ^{1}H,^{1}H-NOESY) of aplysinin A (**2**) in DMSO-d_6

position	δ_C	δ_H	^{1}H,^{1}H-COSY	^{1}H,^{13}C-HMBC	1,1-ADEQ	^{1}H,^{1}H-NOESY
1	73.5	3.93	1-OH	2, 3, 5, 6	---	---
2	113.1	---	---	---	---	---
3	147.1	---	---	---	---	---
4	120.9	---	---	---	---	---
5	131.1	6.56	---	4, 6	6	7
6	90.3	---	---	---	---	---
7	39.7	3.17	---	1, 5, 6, 8, 9	---	5
		3.61	---	---	---	---
8	154.3	--	---	---	---	---
9	159.0	---	---	---	---	---
10	46.3	3.33, 2H	9-NH	9, 12	---	---
11	69.3	4.67	10, 11-OH	10, 12, 13, 17	---	10, 13, 17
12	142.5	---	---	---	---	---
13/17	130.4, 2C	7.57, 2H	---	11, 12, 14/16	12, 16, 14	---
14/16	117.3, 2C	---	---	---	---	---
15	151.4	---	---	---	---	---
18	71.3	3.96, 2H	19	15, 19, 20	---	---
19	29.8	1.91, 2H	20	---	---	---
20	35.7	3.25, 2H	20-NH	21	---	---
21	169.1	---	---	---	---	---
22	22.6	1.80, 3H	---	21	---	---
3-OMe	59.6	3.65, 3H	---	3	---	---
9-NH	---	8.39	10	9	---	11
1-OH	---	6.36	1	1, 2, 6	---	11-OH
11-OH	---	5.72	11	---	---	---
20-NH	---	7.86	20	21	---	---

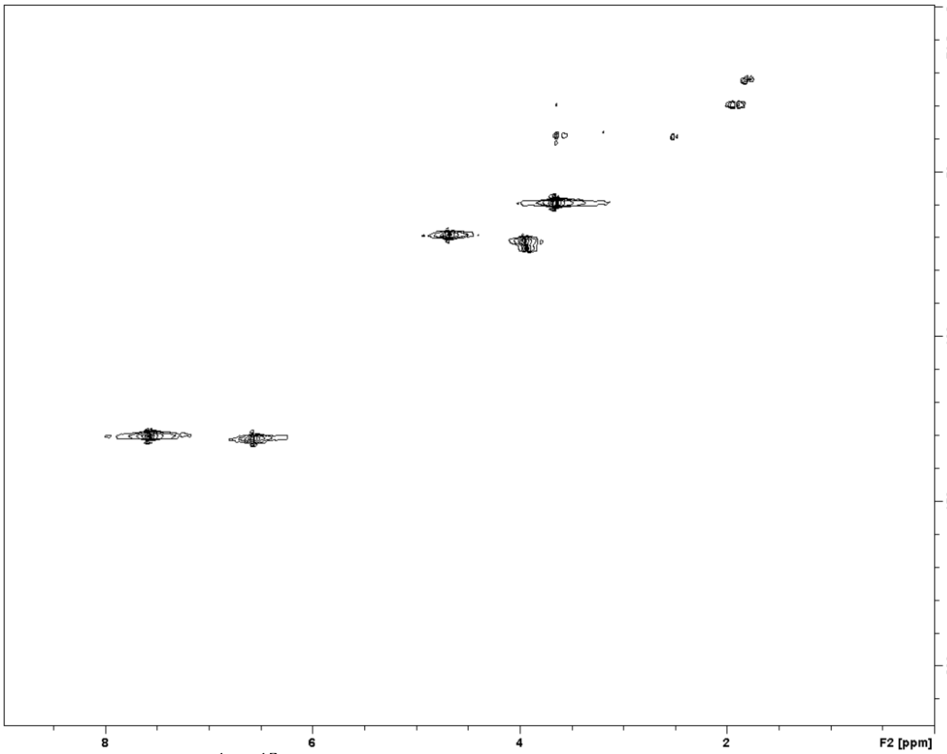

Figure S12: 2D ^{1}H,^{13}C-HSQC spectrum of aplysinin A (**2**) in DMSO- d_6, 303 K.

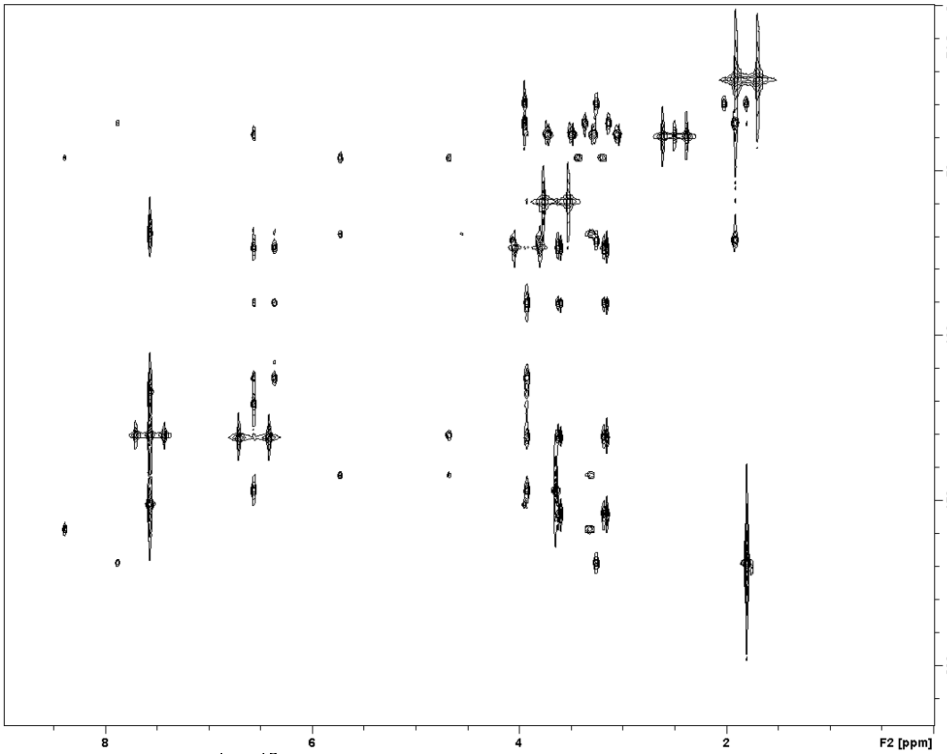

Figure S13: 2D ^{1}H,^{13}C-HMBC spectrum of aplysinin A (**2**) in DMSO- d_6, 303 K.

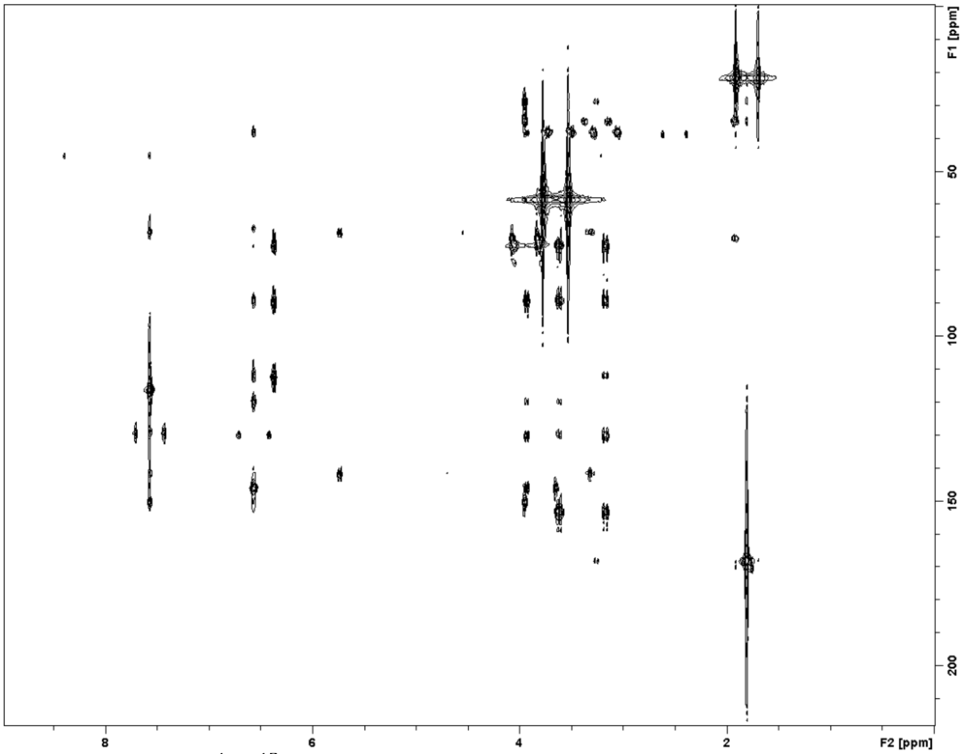

Figure S14: 2D $^{1}H,^{13}C$-HMBC spectrum of aplysinin A (**2**) in DMSO- d_6, D6 = 0.2500, 303 K.

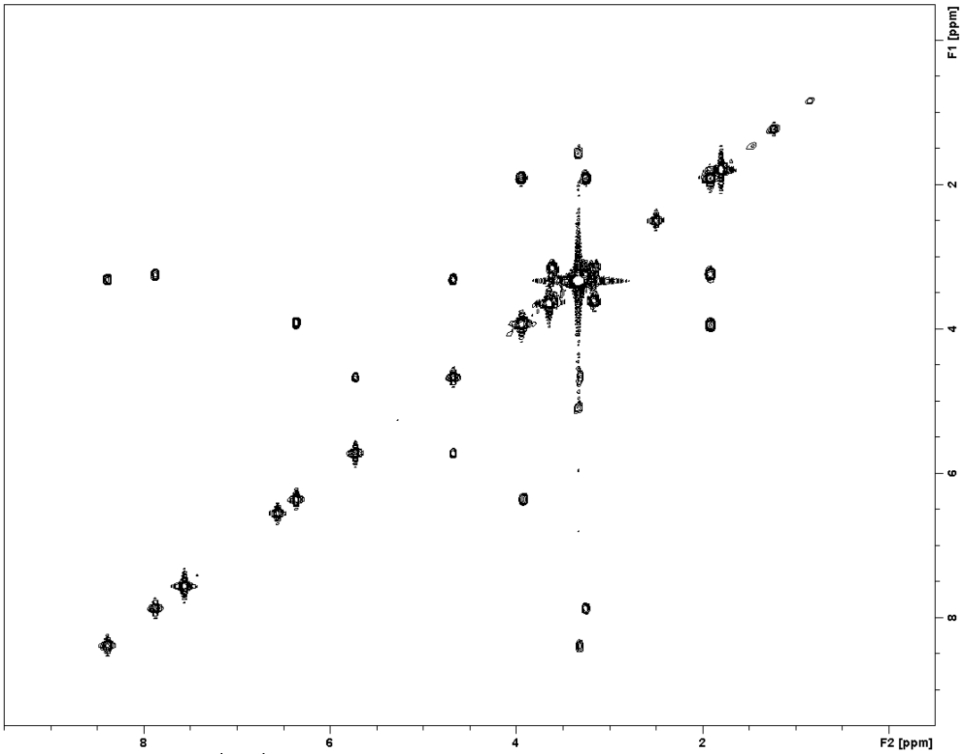

Figure S15: 2D $^{1}H,^{1}H$-COSY spectrum of aplysinin A (**2**) in DMSO- d_6, 303 K.

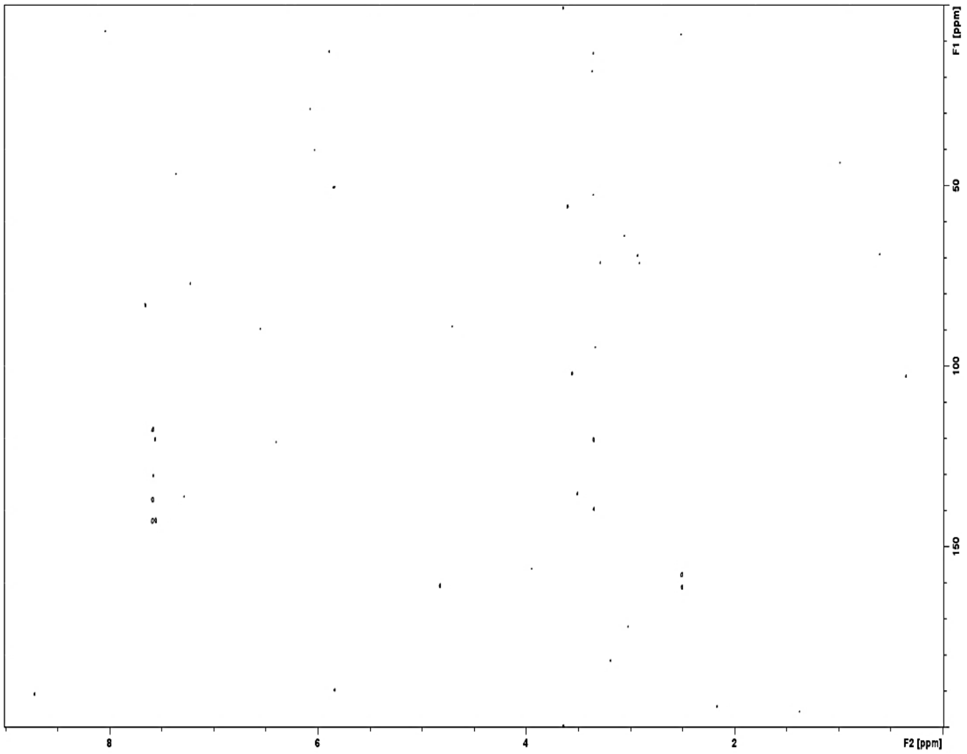

Figure S16: 2D 1,1-ADEQUATE spectrum of aplysinin A (**2**) in DMSO-d_6, 303 K.

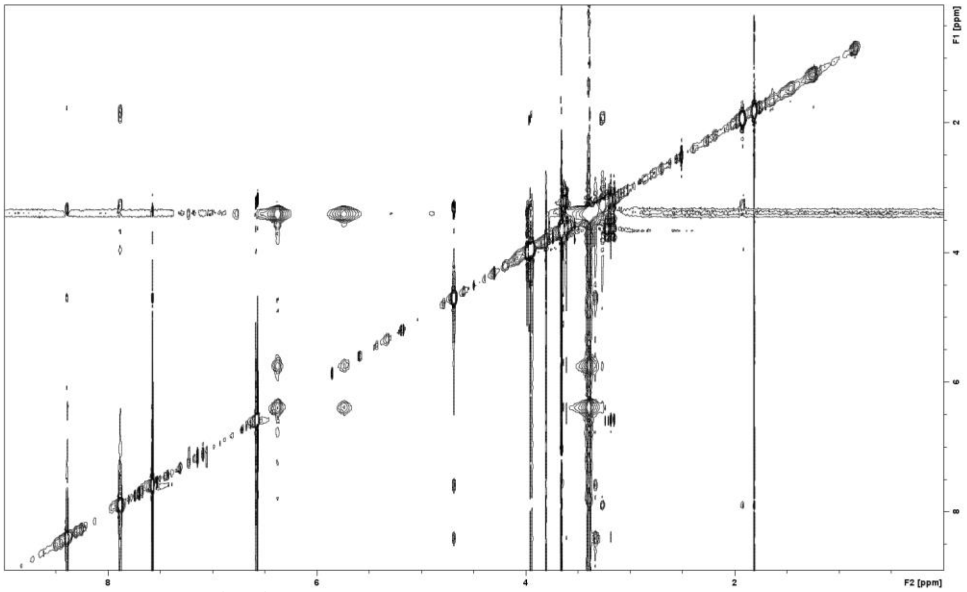

Figure S17: 2D ^{1}H,^{1}H-NOESY spectrum of aplysinin A (**2**) in DMSO-d_6, 303 K.

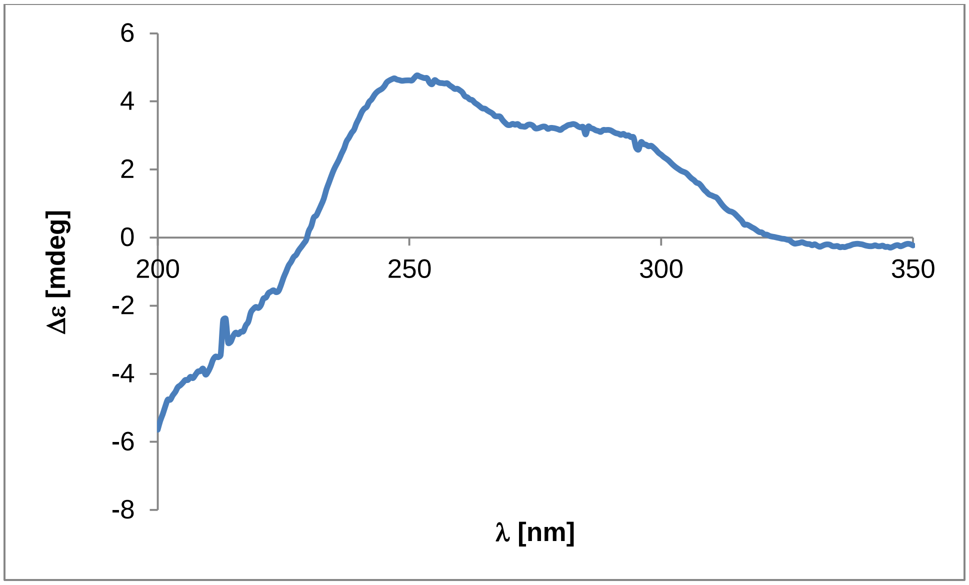

Figure S18: Circular dichroism spectrum of aplysinin A (**2**) ($c = 2.6 \times 10^{-4}$ M, MeOH, 25 °C).

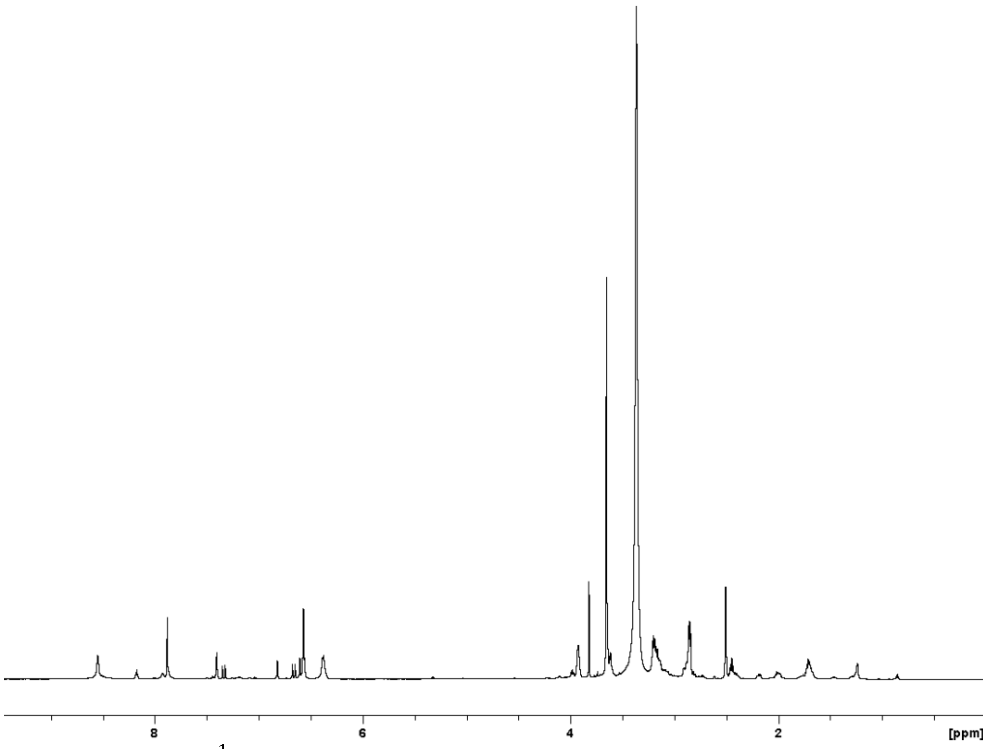

Figure S19: 1D ^{1}H-NMR spectrum of aplysinin B (**3**) in DMSO-d_6, 303 K, 600 MHz.

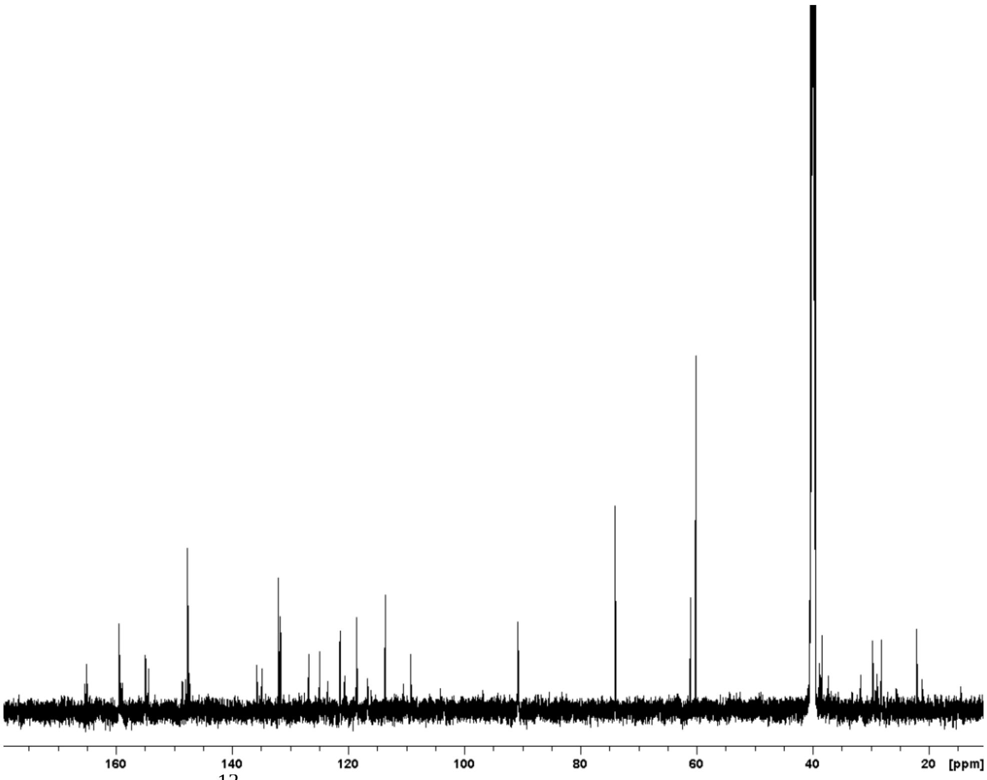

Figure S20: 1D ^{13}C-NMR spectrum of aplysinin B (**3**) in DMSO- d_6, 303 K, 150 MHz.

Table S3: NMR data (^{13}C, ^{1}H, ^{1}H,^{1}H-COSY, and ^{1}H,^{13}C-HMBC) of aplysinin B (**3**) in DMSO-d_6

position	δ_C	δ_H	^{1}H,^{1}H-COSY	^{1}H,^{13}C-HMBC
1	134.9	---	---	---
2/6	131.7, 2C	7.88, 2H	---	3, 4, 5, 7
3/5	118.5, 2C	---	---	---
4	154.4	---	---	---
7	135.7	7.33	---	1, 2, 6, 8, 9
8	124.9	6.66	---	1, 9
9	165.0	---	---	---
10	38.4	3.20	---	9, 11
11	28.1	1.71, 2H	11	10, 12, 13
12	22.0	2.45, 2H	12	13, 14
13	126.8	---	---	---
14	109.2	6.60	---	13, 15
15	147.4	---	---	---
4-OMe	61.0	3.82, 3H	---	4
9-NH	---	8.17	10	9, 10

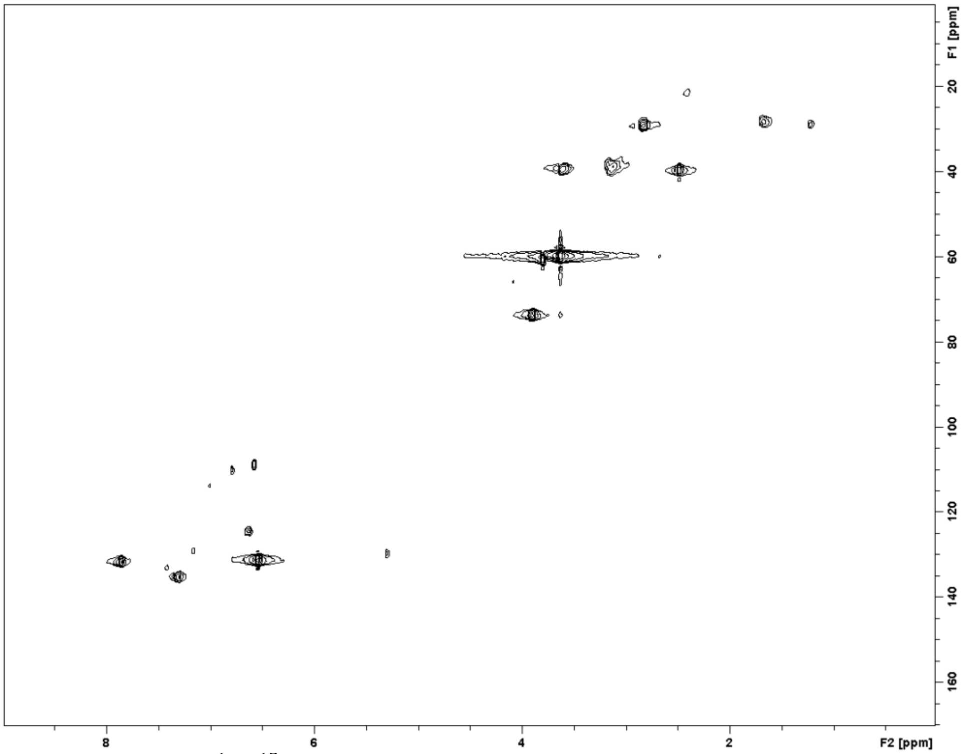

Figure S21: 2D ^{1}H,^{13}C-HSQC spectrum of aplysinin B (**3**) in DMSO-d_6, 303 K.

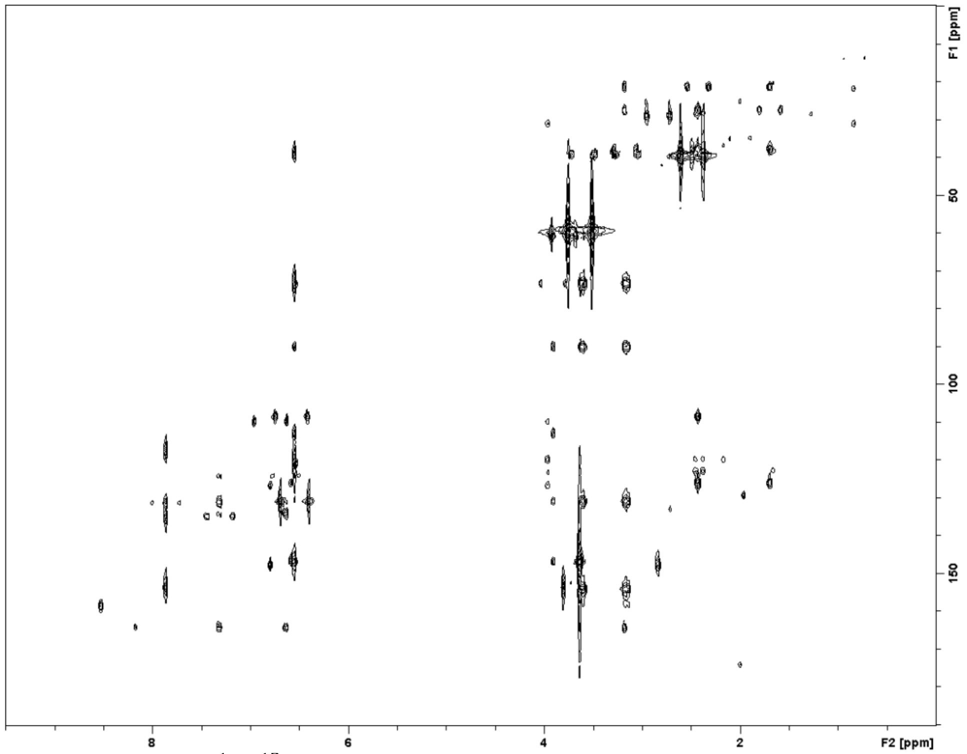

Figure S22: 2D ^{1}H,^{13}C-HMBC spectrum of aplysinin B (**3**) in DMSO-d_6, 303 K.

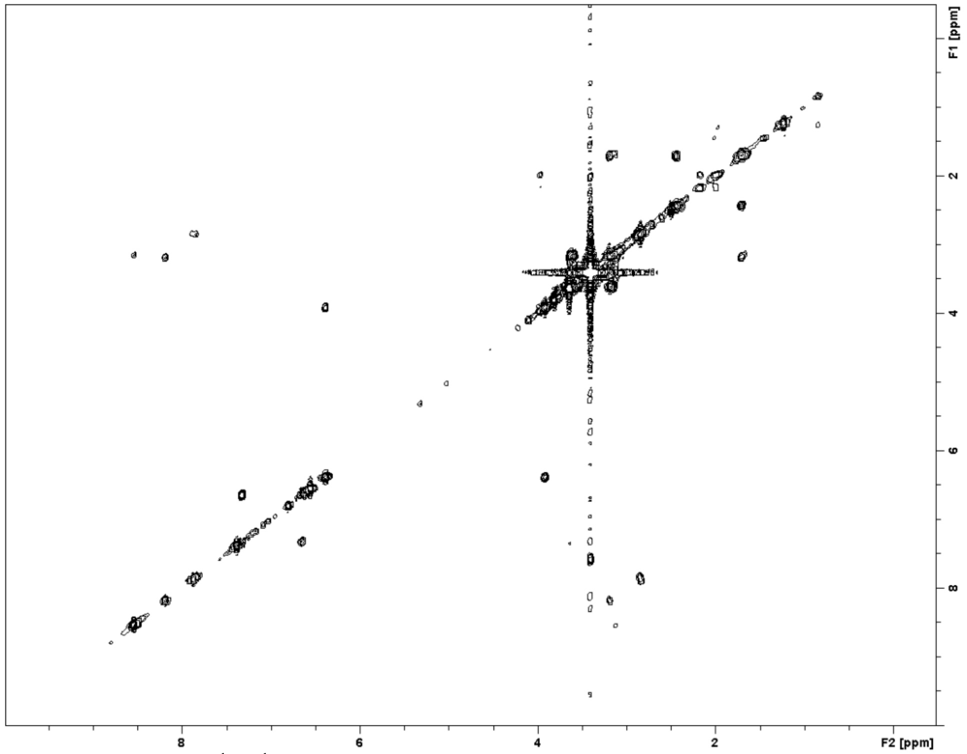

Figure S23: 2D ^{1}H,^{1}H-COSY spectrum of aplysinin B (**3**) in DMSO-d_6, 303 K.

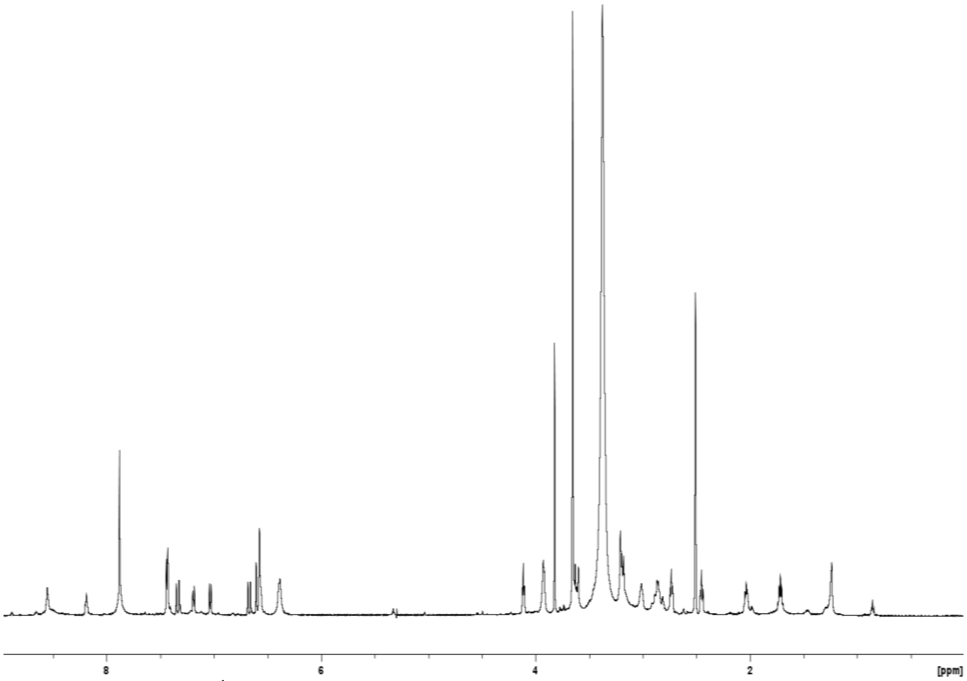

Figure S24: 1D ^{1}H-NMR spectrum of 14-debromoaraplysillin I (**4**) in DMSO-d_6, 303 K, 600 MHz.

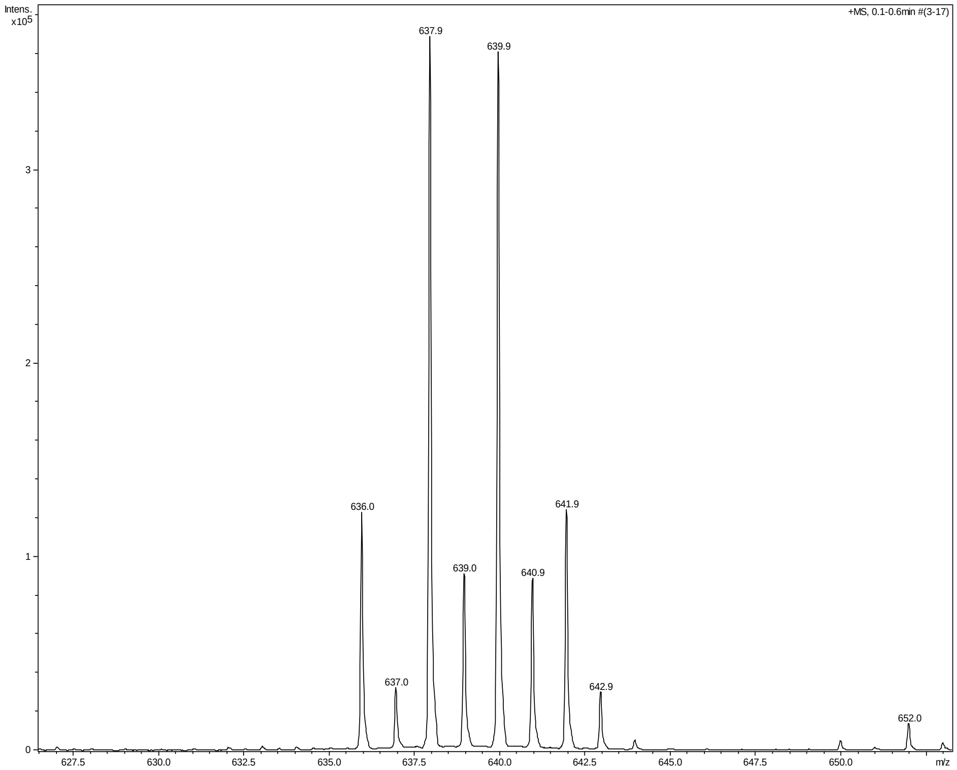

Figure S25: ESI–MS(+) spectral data of 14-debromoaraplysillin I (**4**).

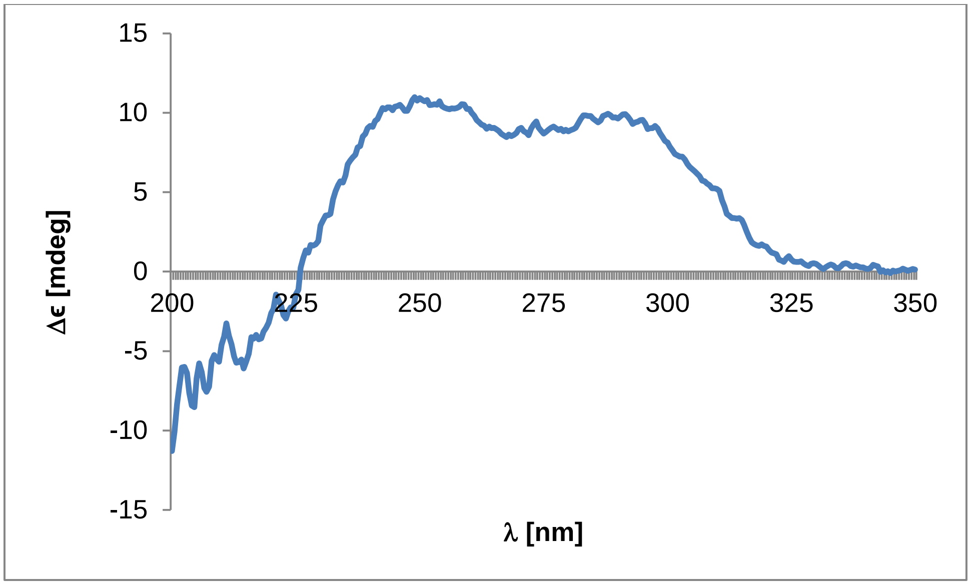

Figure S26: Circular dichroism spectrum of 14-debromoaraplysillin I (**4**) ($c = 3.1 \times 10^{-4}$ M, MeOH, 25 °C).

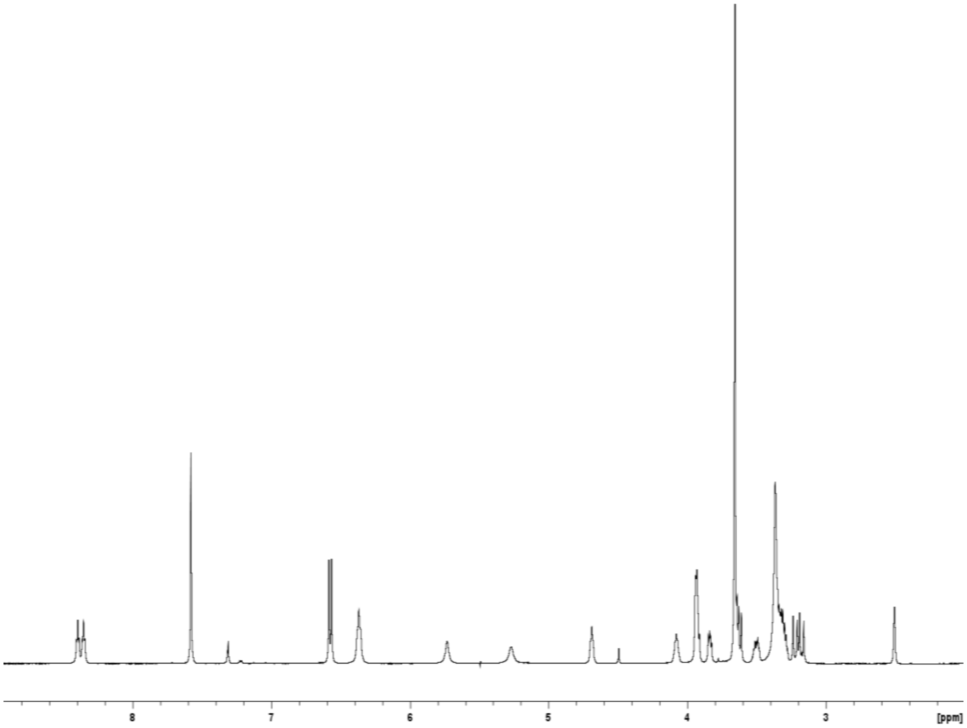

Figure S27: 1D ^{1}H-NMR spectrum of fistularin-3 (**5**) in DMSO-d_6, 303 K, 600 MHz.

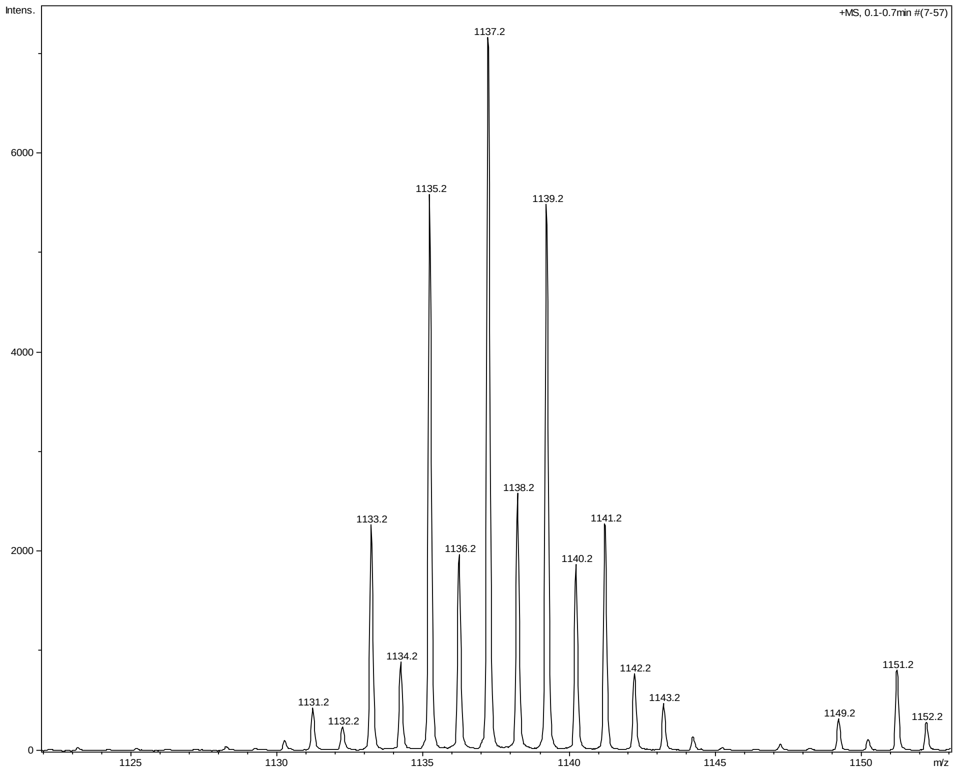

Figure S28: ESI–MS(+) spectral data of fistularin-3 (**5**).

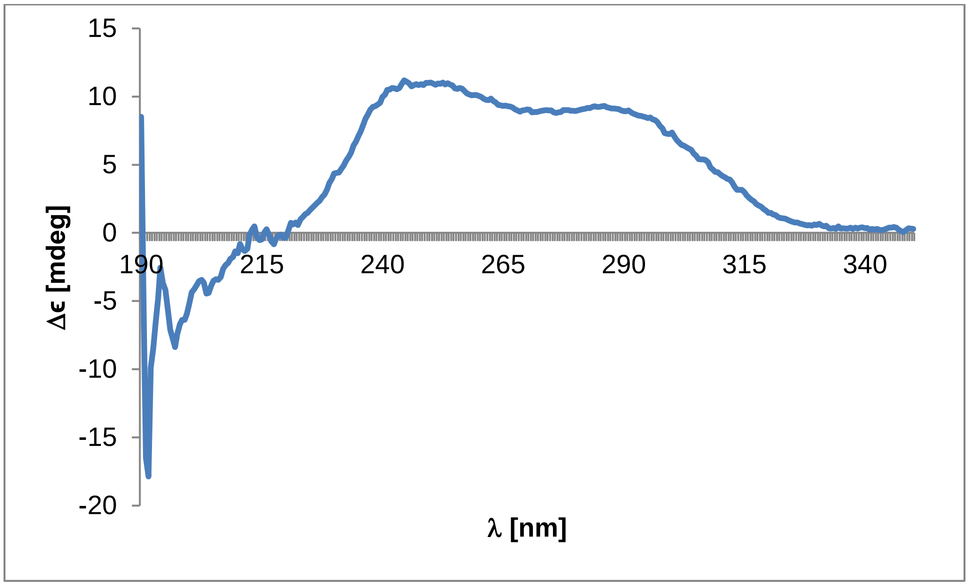

Figure S29: Circular dichroism spectrum of fistularin-3 (**5**) ($c = 1.7 \times 10^{-4}$ M, MeOH, 25 °C).

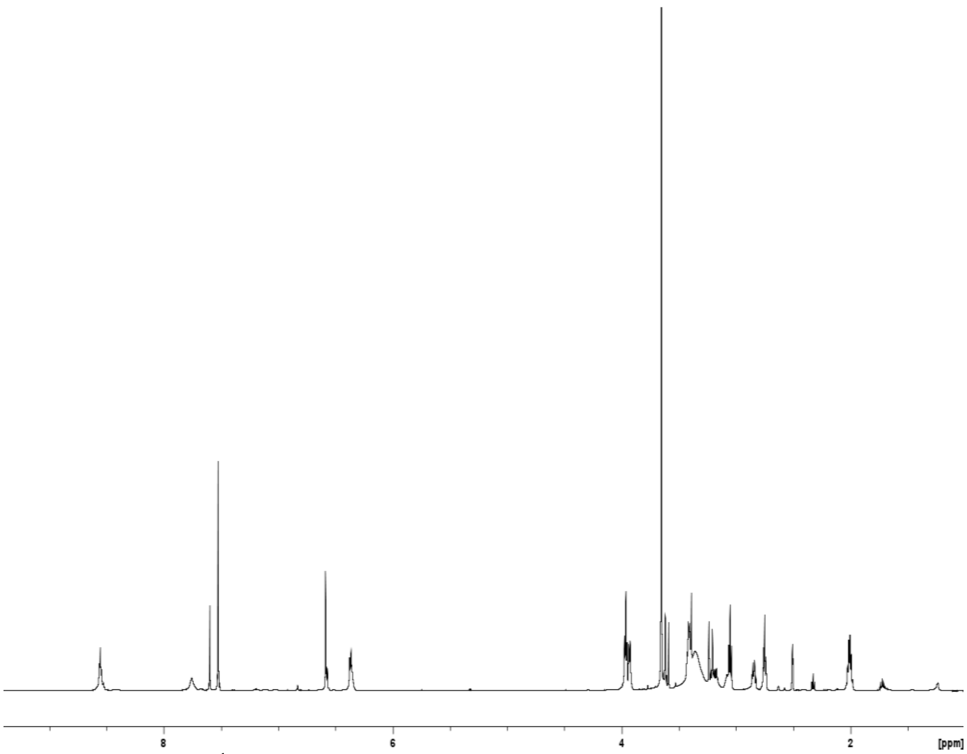

Figure S30: 1D ^{1}H-NMR spectrum of 11,19-dideoxyfistularin-3 (**6**) in DMSO-d_6, 303 K, 600 MHz.

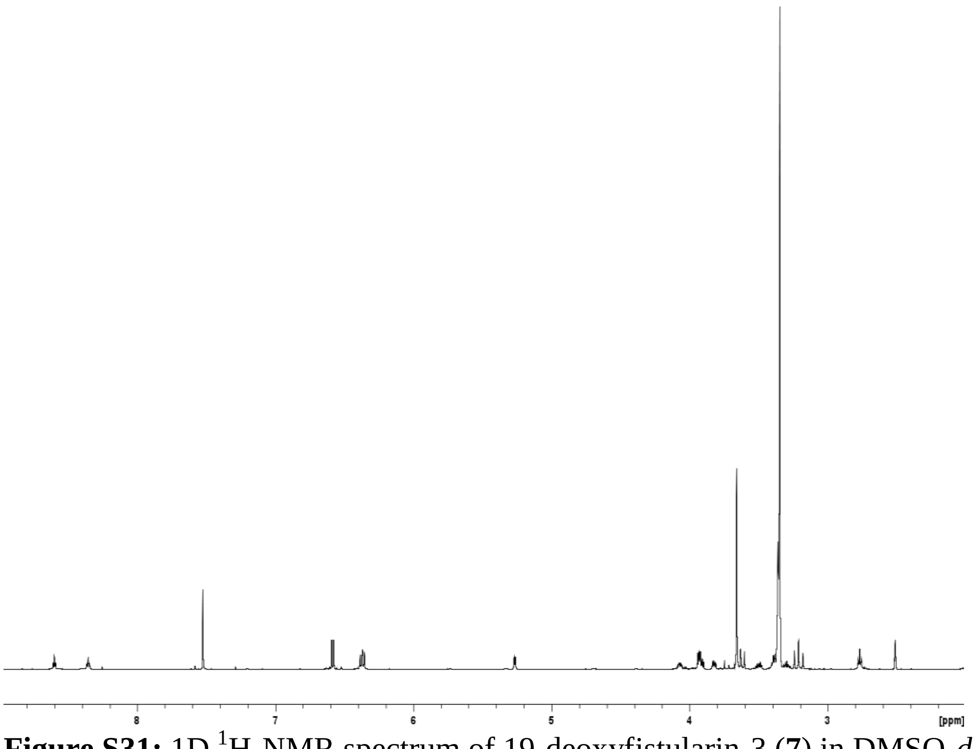

Figure S31: 1D ^{1}H-NMR spectrum of 19-deoxyfistularin-3 (**7**) in DMSO-d_6, 303 K, 600 MHz.

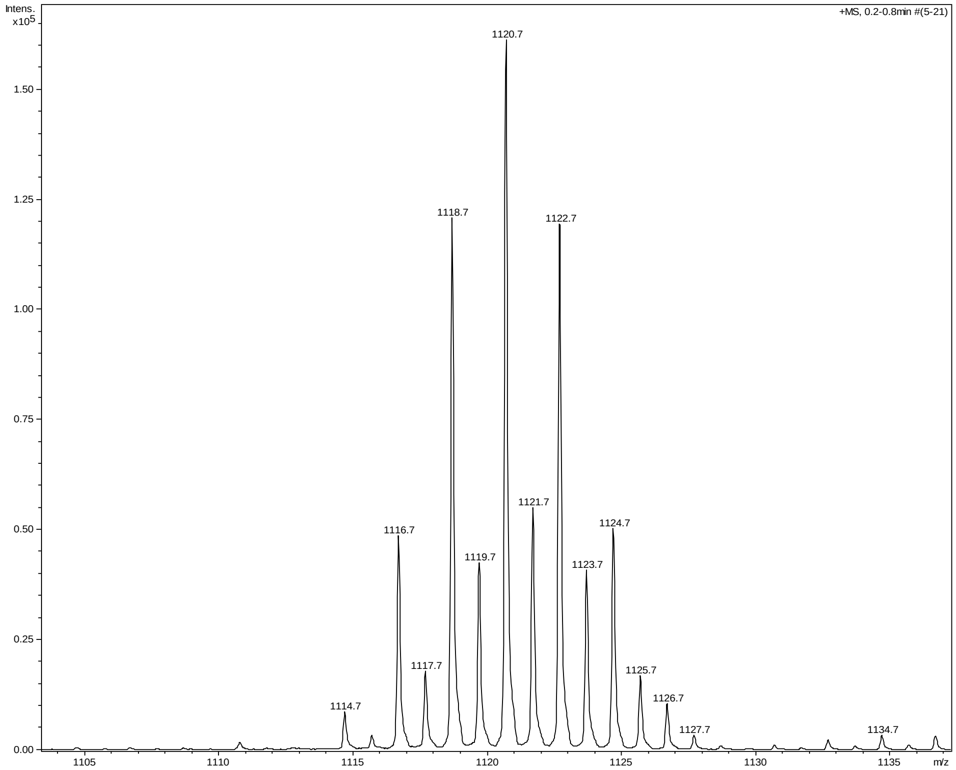

Figure S32: ESI–MS(+) spectral data of 19-deoxyfistularin-3 (**7**).

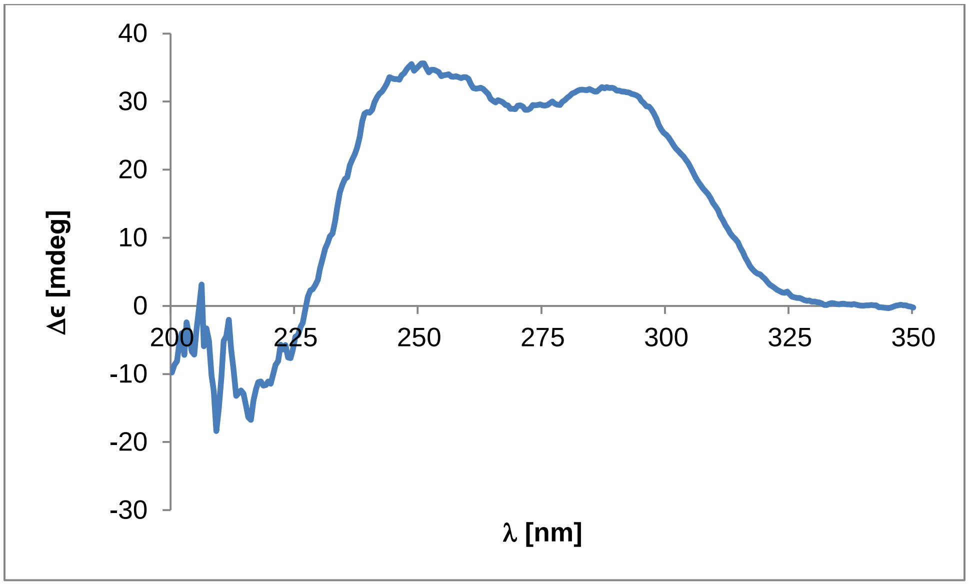

Figure S33: Circular dichroism spectrum of 19-deoxyfistularin-3 (**7**) ($c = 1.8 \times 10^{-4}$ M, MeOH, 25 °C).

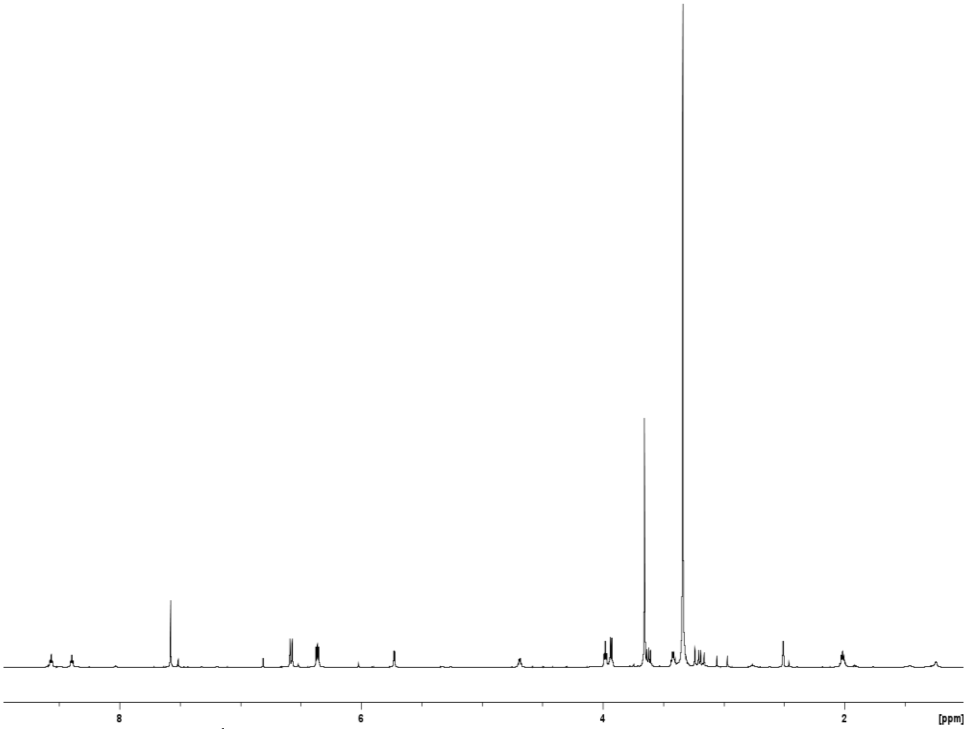

Figure S34: 1D ^{1}H-NMR spectrum of 11-deoxyfistularin-3 (**8**) in DMSO-d_6, 303 K, 600 MHz.

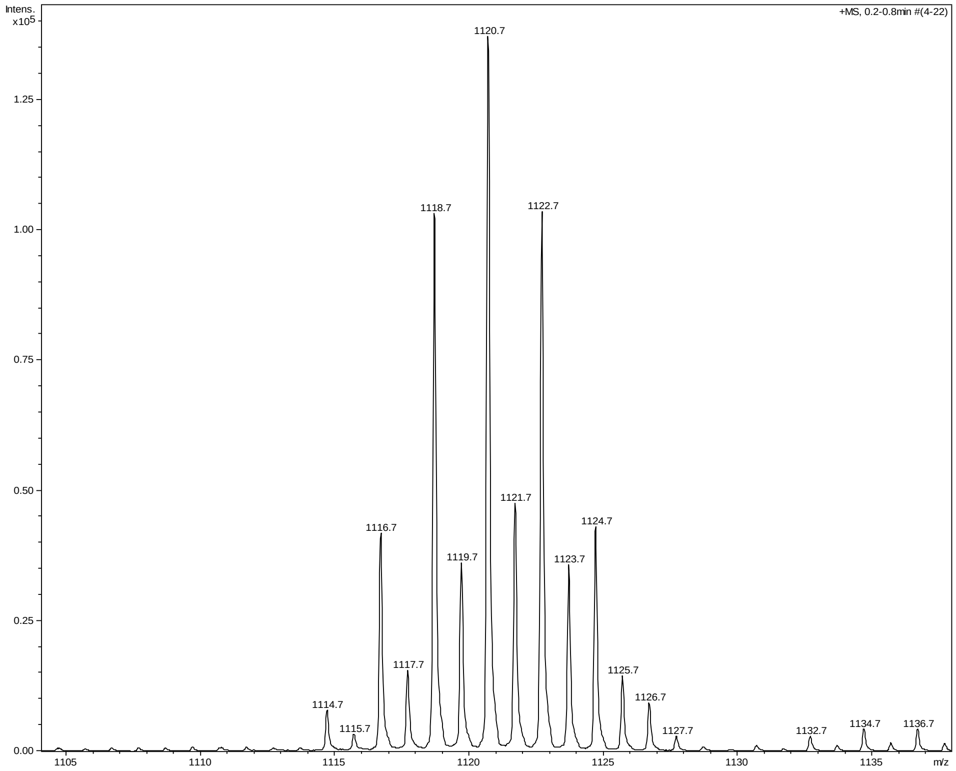

Figure S35: ESI–MS(+) spectral data of 11-deoxyfistularin-3 (**8**).

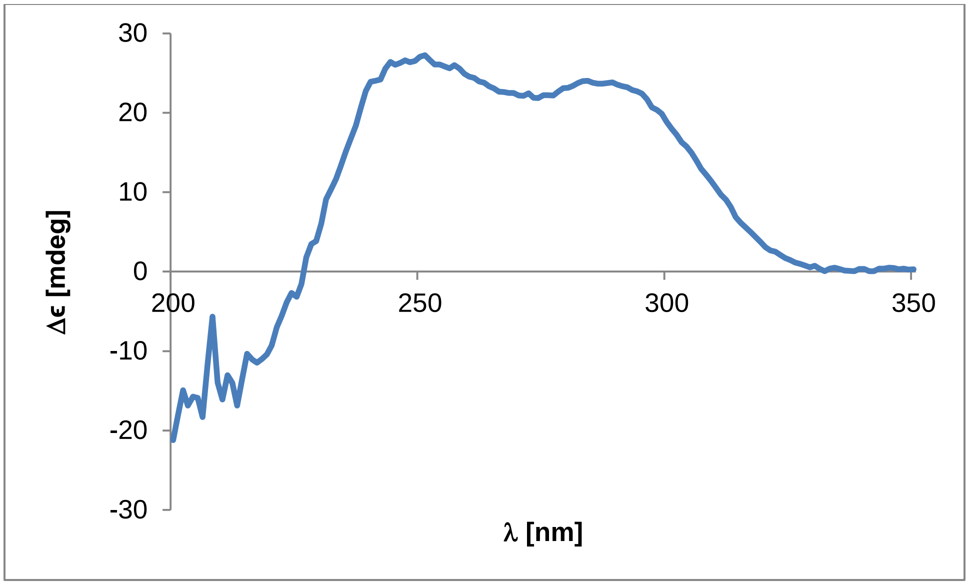

Figure S36: Circular dichroism spectrum of 11-deoxyfistularin-3 (**8**) ($c = 1.8 \times 10^{-4}$ M, MeOH, 25 °C).

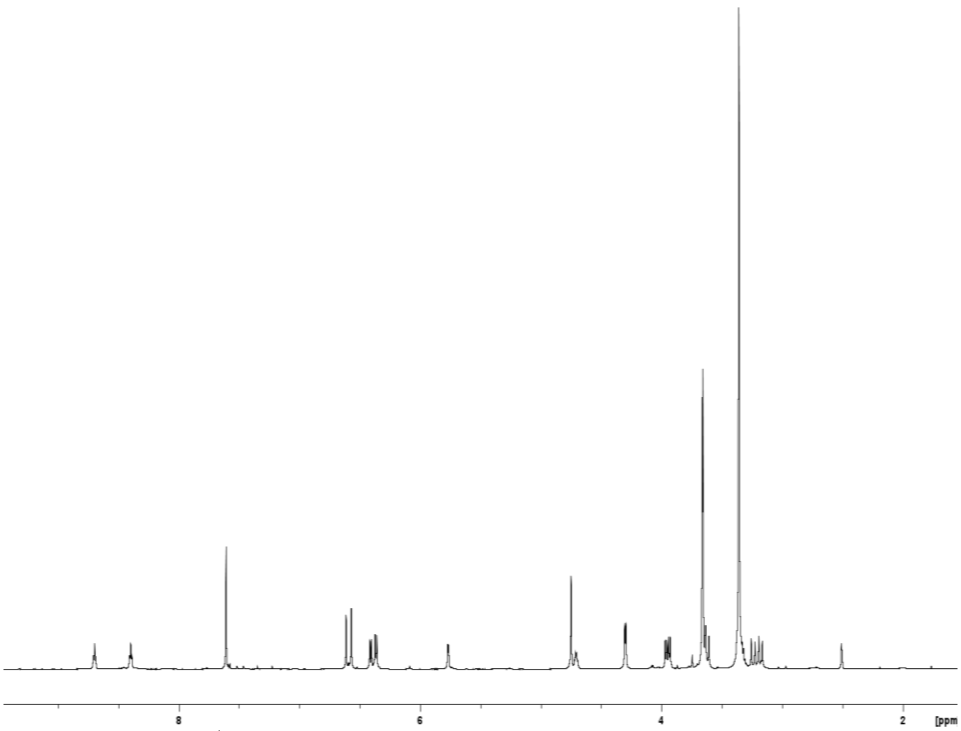

Figure S37: 1D ^{1}H-NMR spectrum of 11-ketofistularin-3 (**9**) in DMSO-d_6, 303 K, 600 MHz.

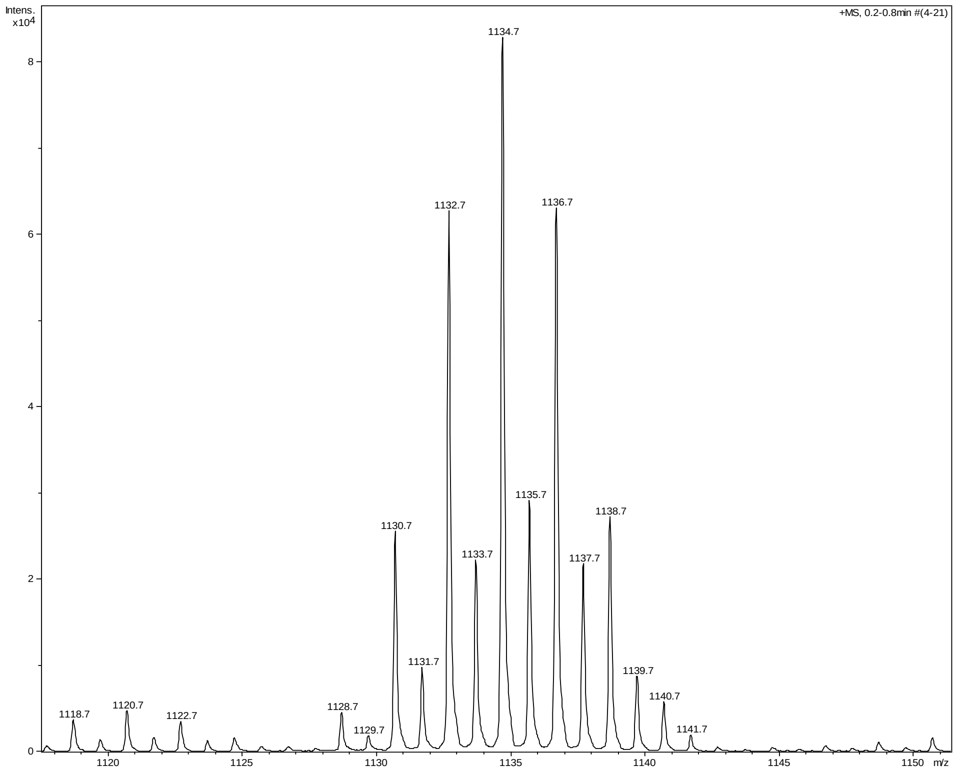

Figure S38: ESI–MS(+) spectral data of 11-ketofistularin-3 (**9**).

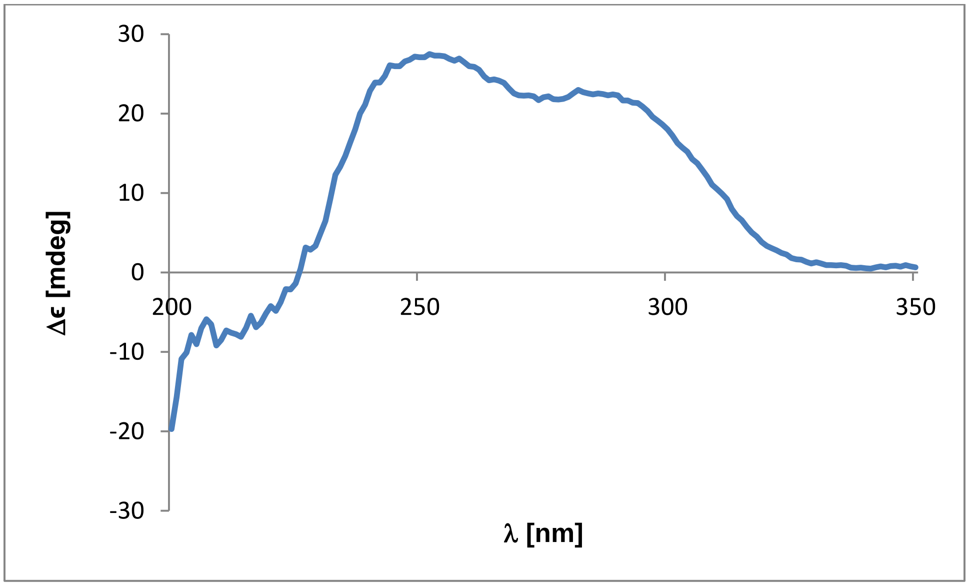

Figure S39: Circular dichroism spectrum of 11-ketofistularin-3 (**9**) ($c = 1.8 \times 10^{-4}$ M, MeOH, 25 °C).

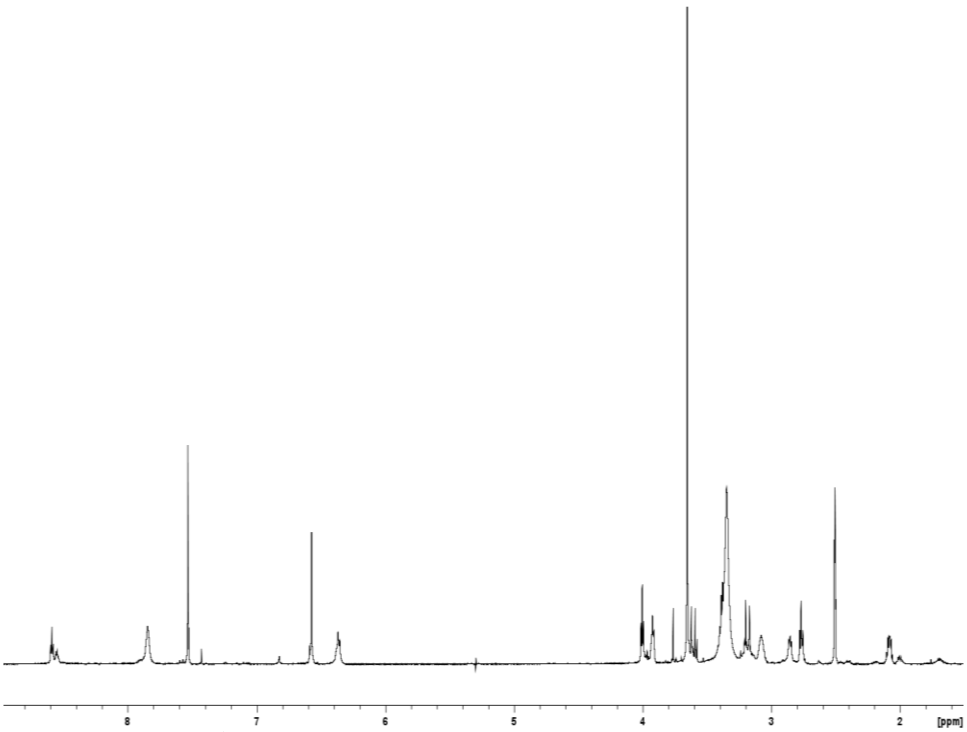

Figure S40: 1D ^{1}H-NMR spectrum of hexadelin B (**10**) in DMSO-d_6, 303 K, 600 MHz.

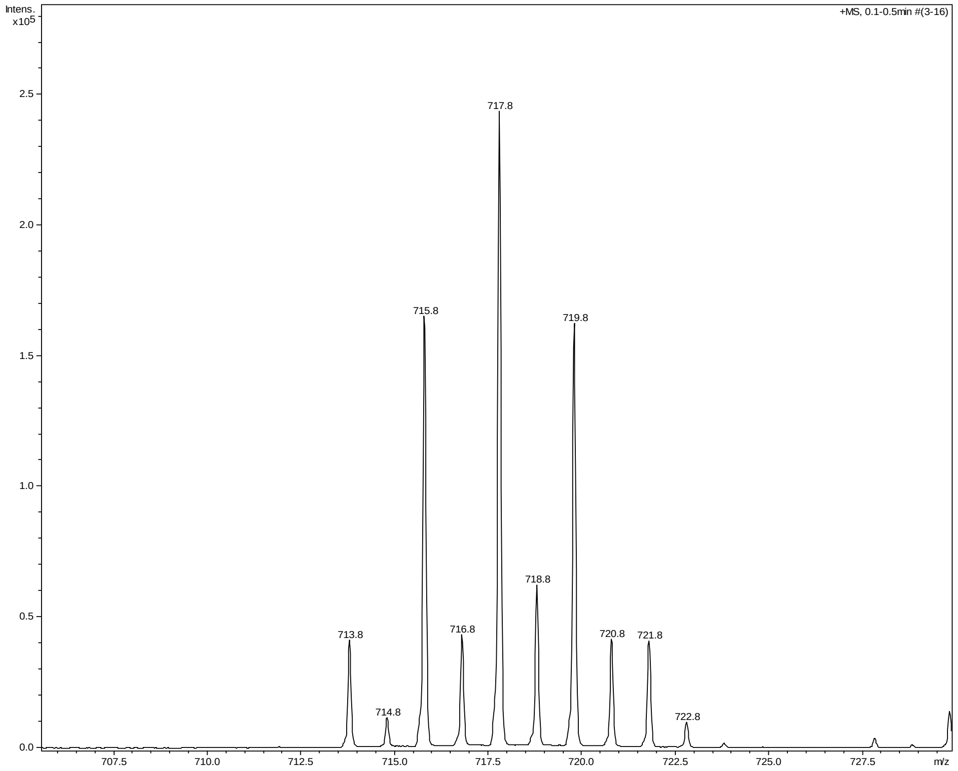

Figure S41: ESI–MS(+) spectral data of hexadelin B (**10**).

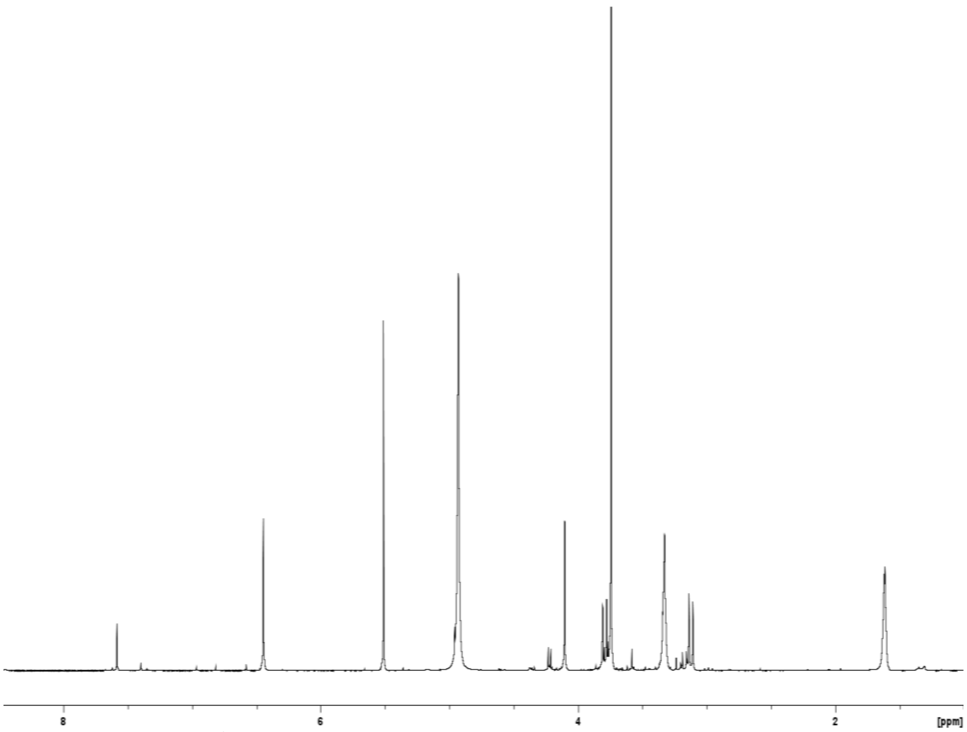

Figure S42: 1D ^{1}H-NMR spectrum of aerothionin (**11**) in DMSO-d_6, 303 K, 600 MHz.

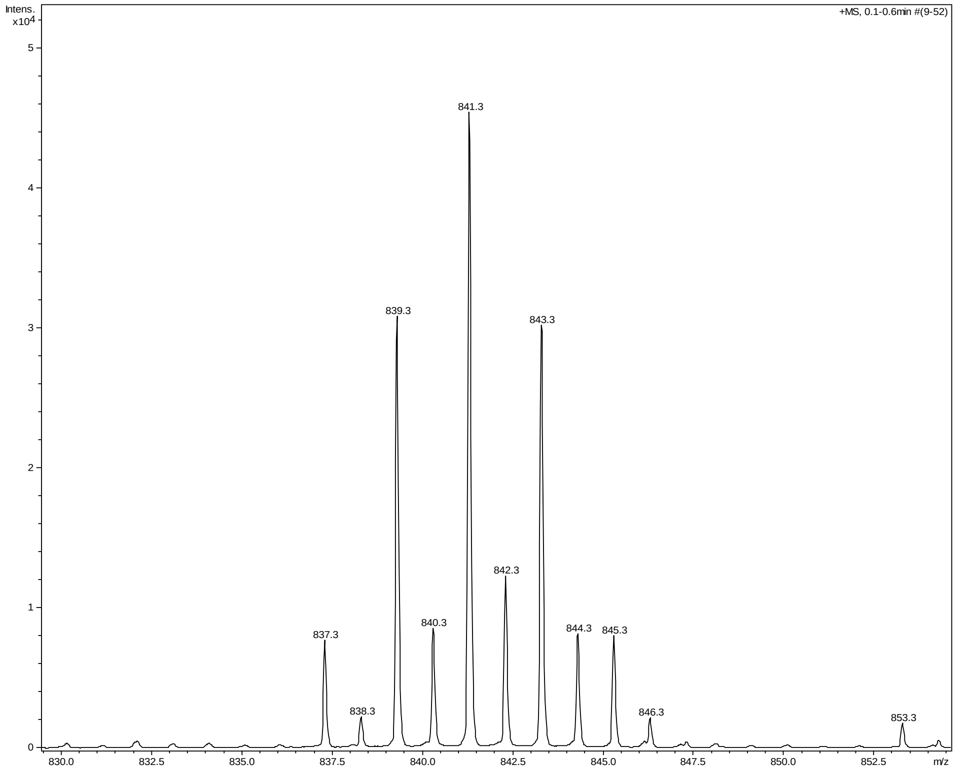

Figure S43: ESI–MS(+) spectral data of aerothionin (**11**).

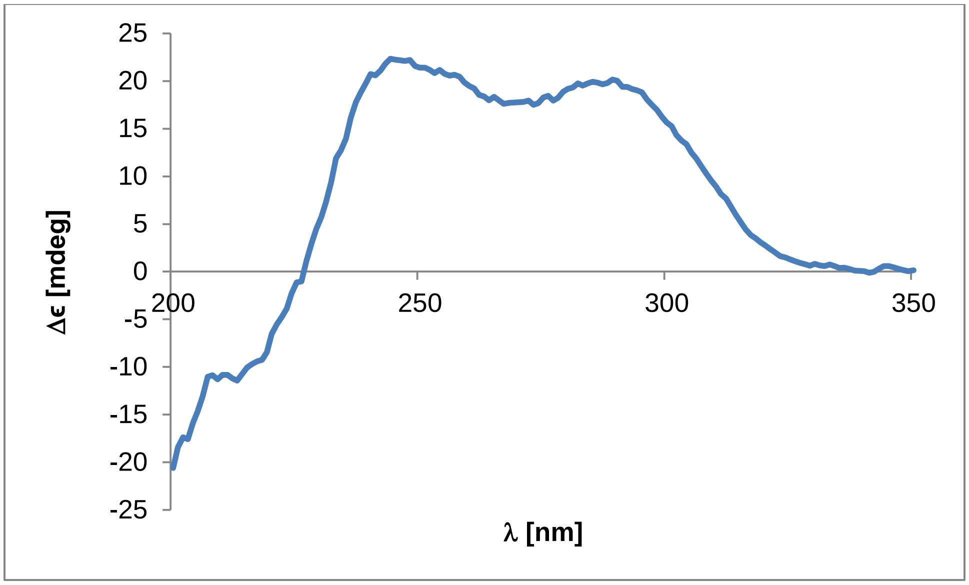

Figure S44: Circular dichroism spectrum of aerothionin (**11**) (c = 2.4 × 10^{-4} M, MeOH, 25 °C).

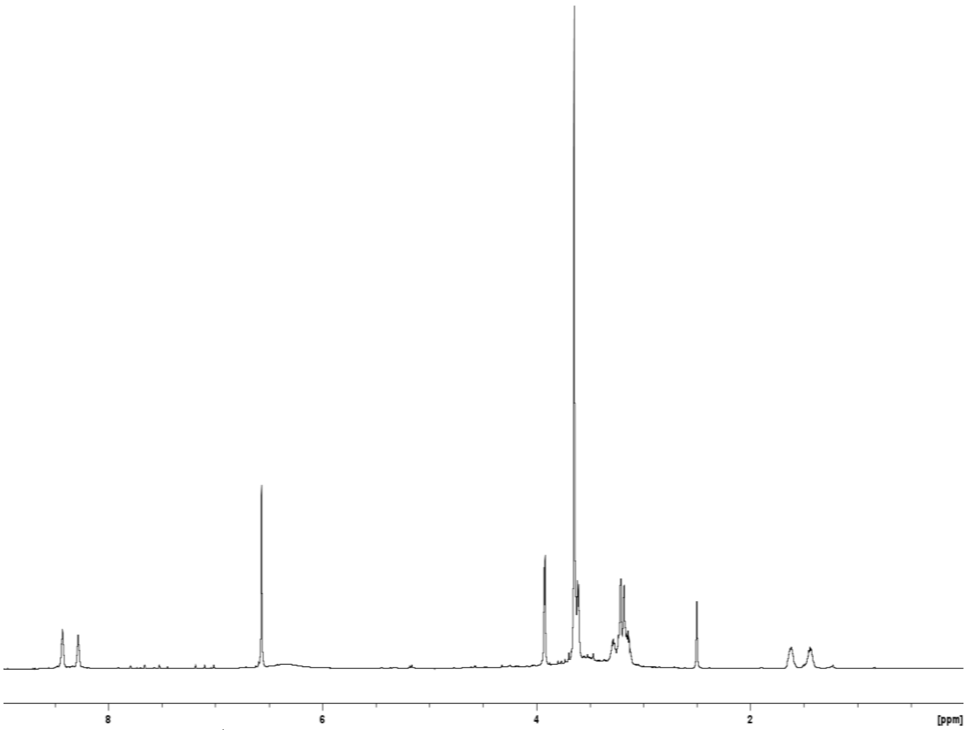

Figure S45: 1D ^{1}H-NMR spectrum of 11-hydroxyaerothionin (**12**) in DMSO-d_6, 303 K, 600 MHz.

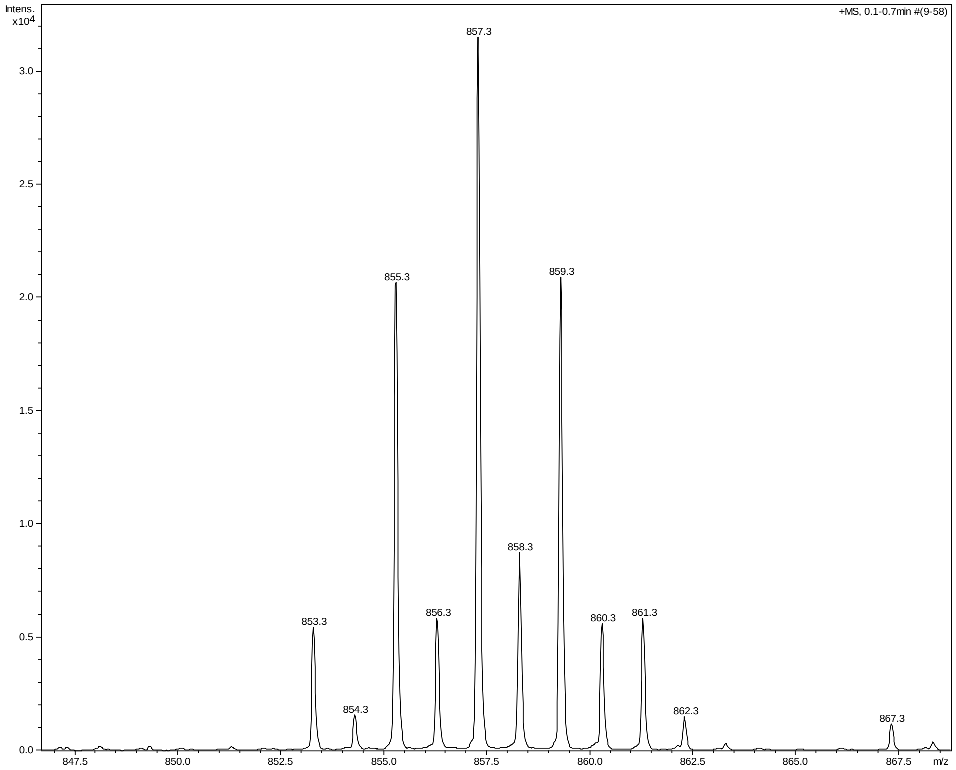

Figure S46: ESI–MS(+) spectral data of 11-hydroxyaerothionin (**12**).

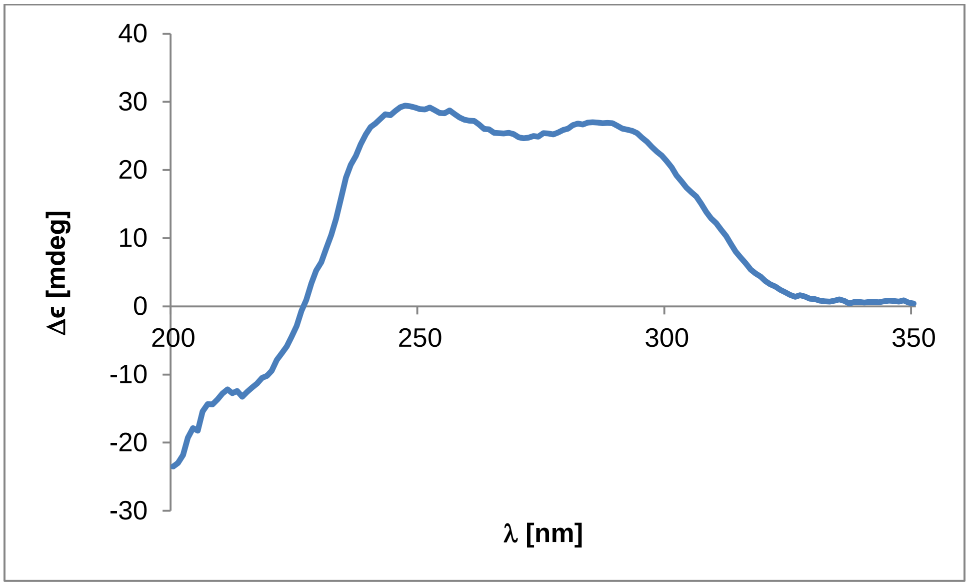

Figure S47: Circular dichroism spectrum of 11-hydroxyaerothionin (**12**) (c = 2.4 × 10^{-4} M, MeOH, 25 °C).

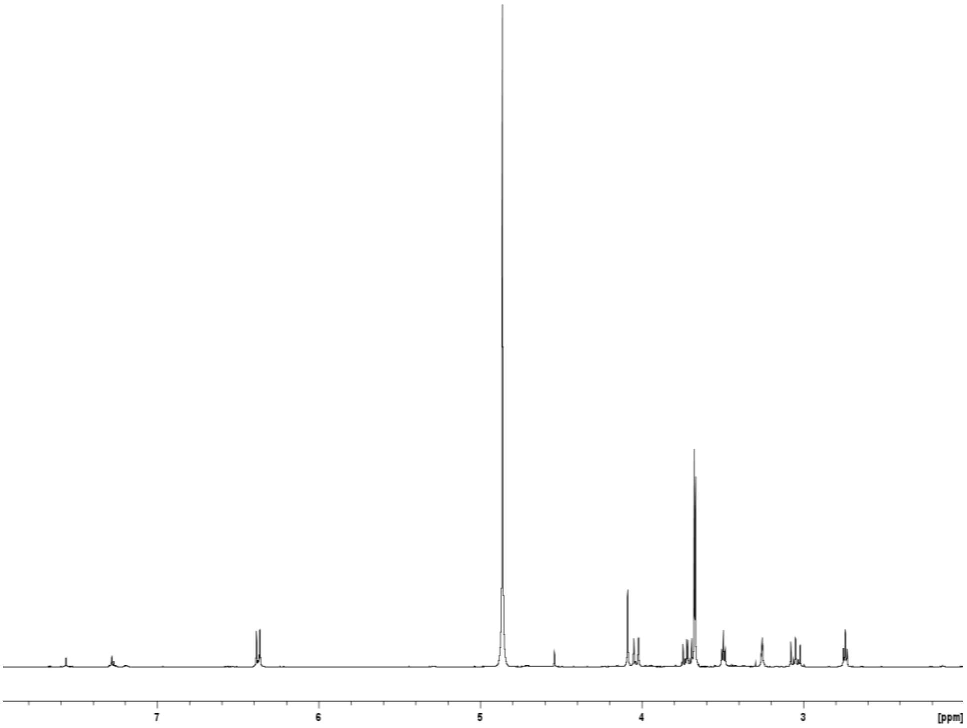

Figure S48: 1D ^{1}H-NMR spectrum of 11-oxoaerothionin (**13**) in CD_3OD, 303 K, 600 MHz.

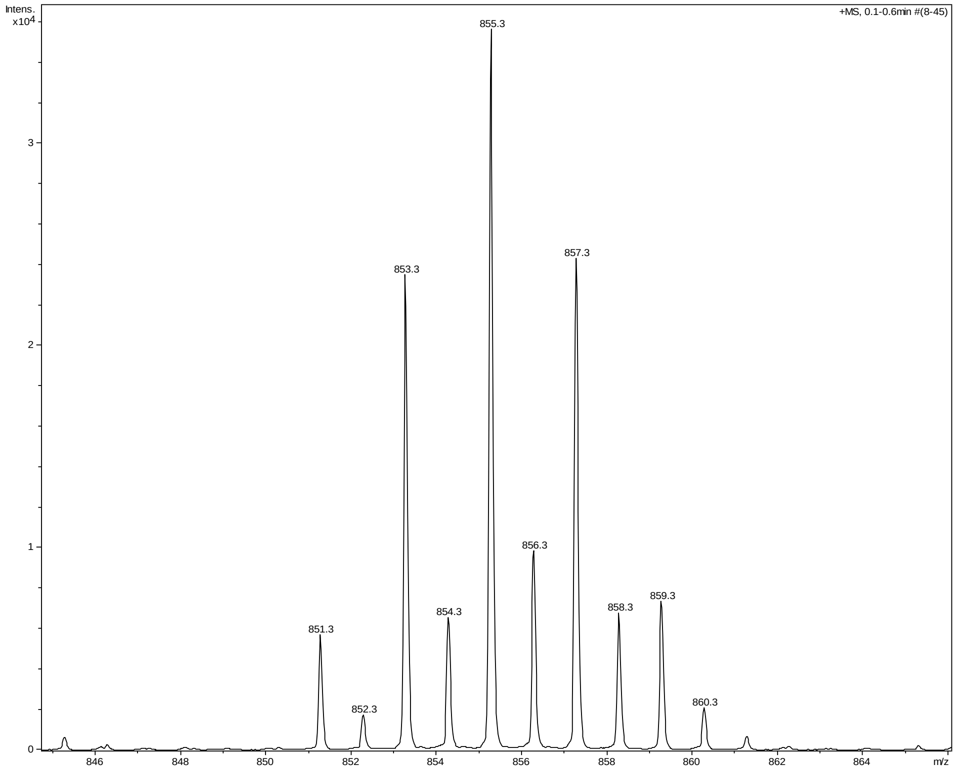

Figure S49: ESI–MS(+) spectral data of 11-oxoaerothionin (**13**).

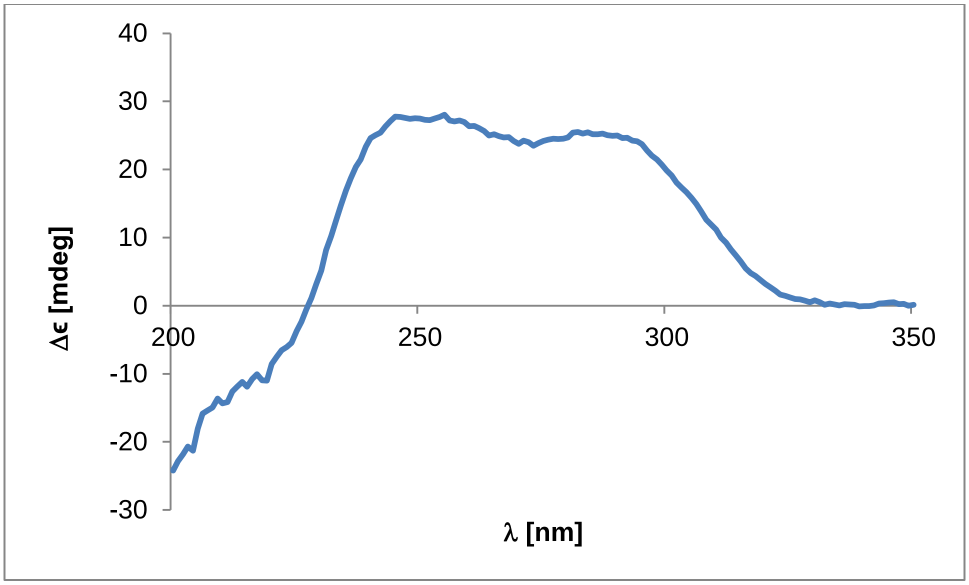

Figure S50: Circular dichroism spectrum of 11-oxoaerothionin (**13**) ($c = 2.4 \times 10^{-4}$ M, MeOH, 25 °C).

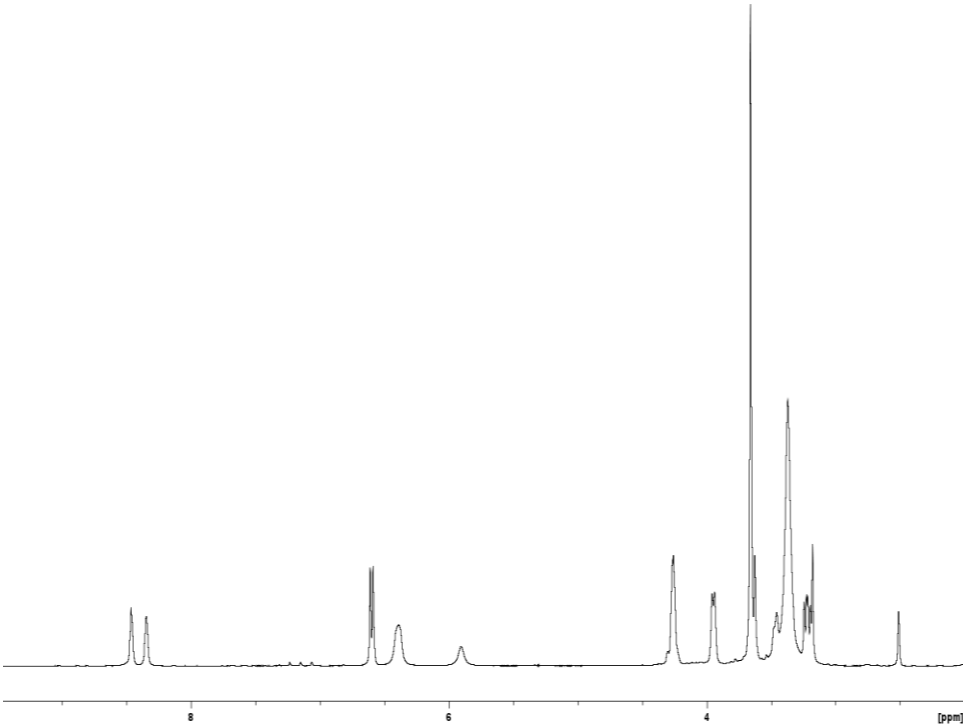

Figure S51: 1D ^{1}H-NMR spectrum of 11-oxo-12-hydroxyaerothionin (**14**) in DMSO-d_6, 303 K, 600 MHz.

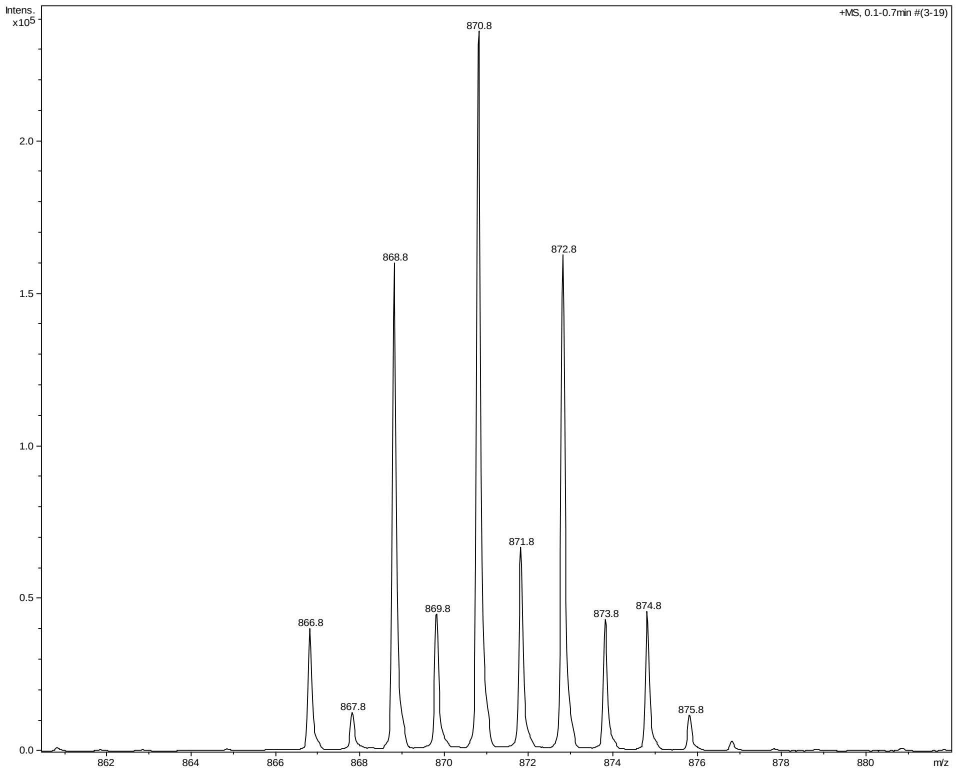

Figure S52: ESI–MS(+) spectral data of 11-oxo-12-hydroxyaerothionin (**14**).

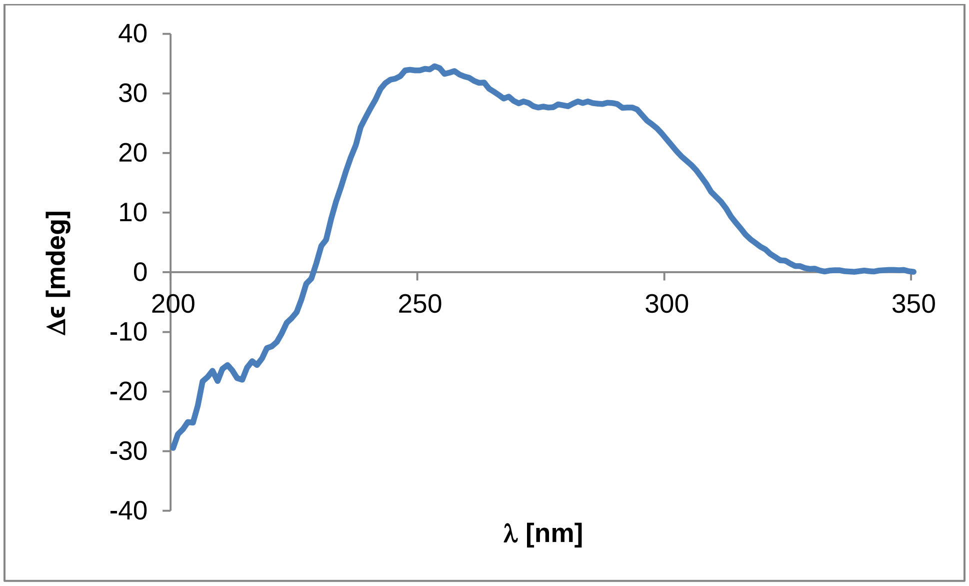

Figure S53: Circular dichroism spectrum of 11-oxo-12-hydroxyaerothionin (**14**) (c = 2.3 × 10^{-4} M, MeOH, 25 °C)

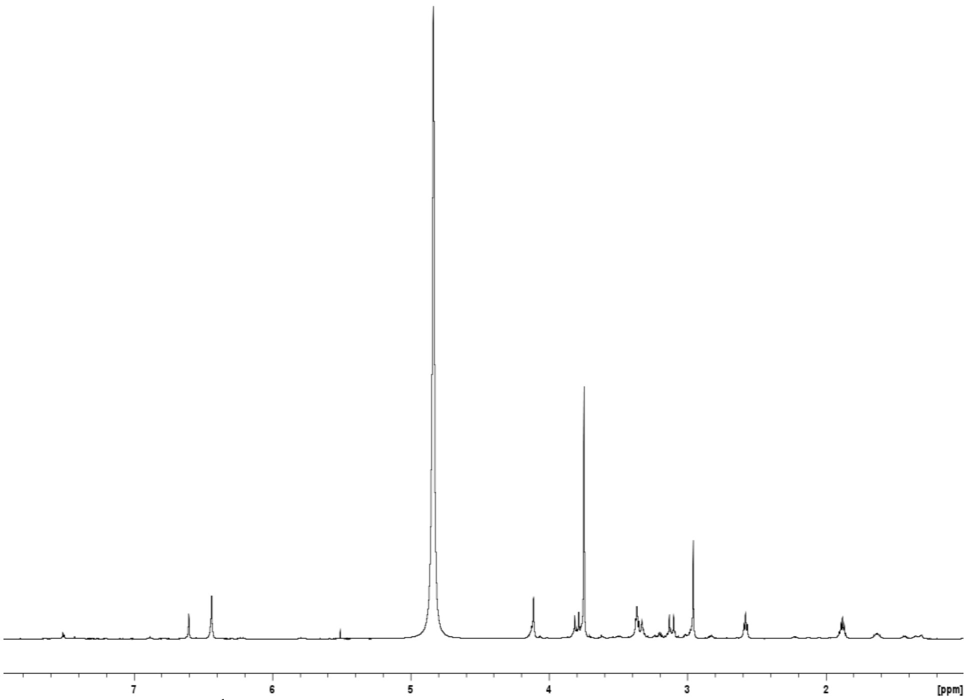

Figure S54: 1D ^{1}H-NMR spectrum of *N*-methyl-aerophobin-2 (**15**) in CD_3OD-d_4, 303 K, 600 MHz.

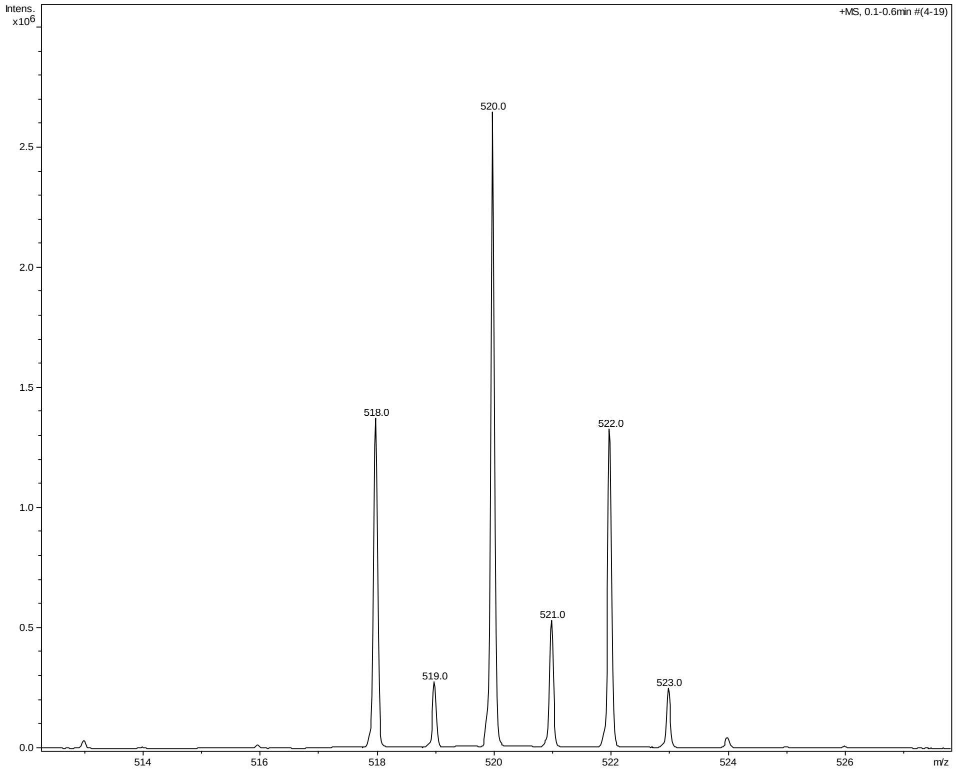

Figure S55: ESI–MS(+) spectral data of *N*-methyl-aerophobin-2 (**15**).

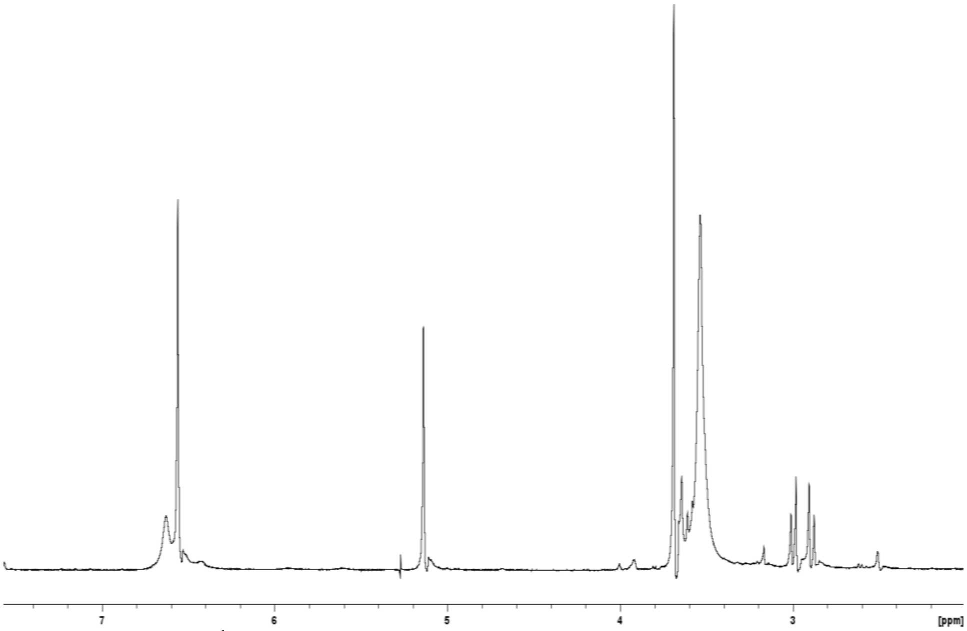

Figure S56: 1D ^{1}H-NMR spectrum of aeroplysinin-2 (**16**) in DMSO-d_6, 303 K, 600 MHz.

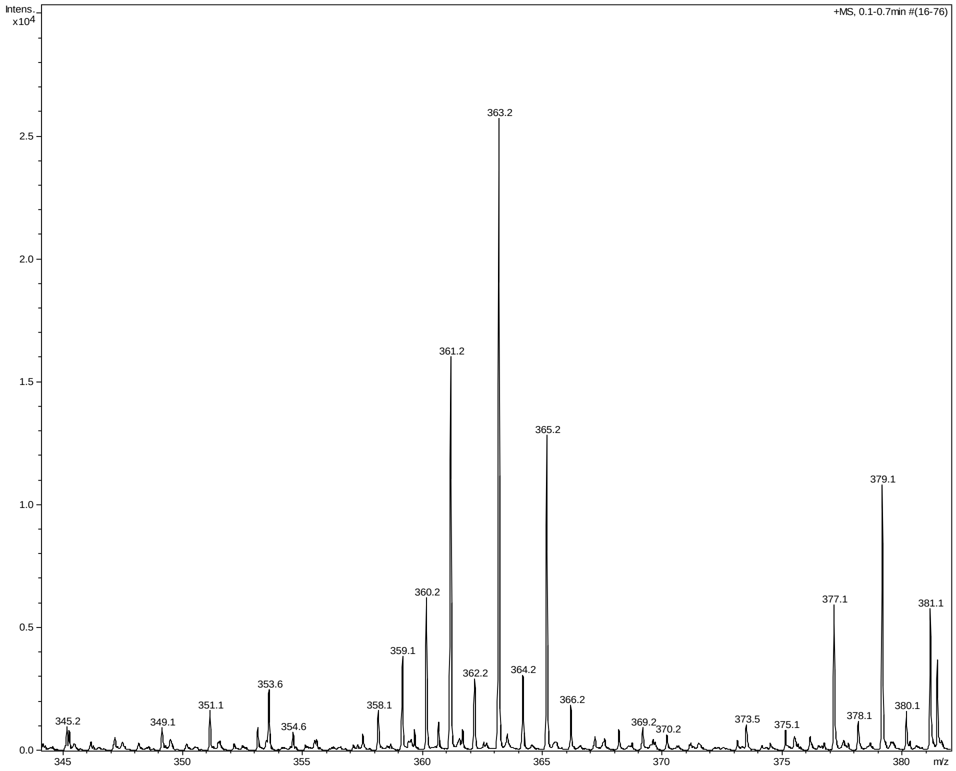

Figure S57: ESI–MS(+) spectral data of aeroplysinin-2 (**16**).

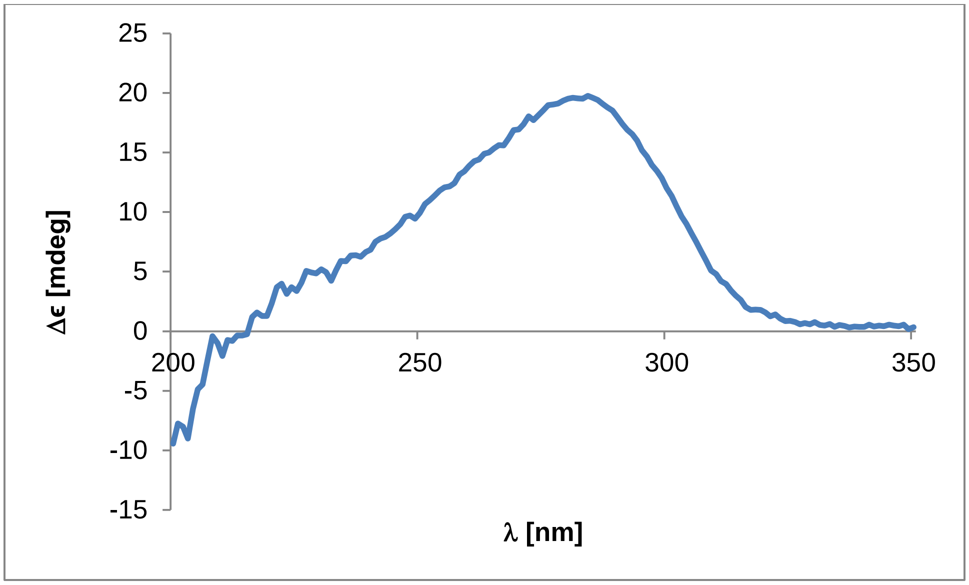

Figure S58: Circular dichroism spectrum of aeroplysinin-2 (**16**) (c = 5.5 × 10^{-4} M, MeOH, 25 °C)

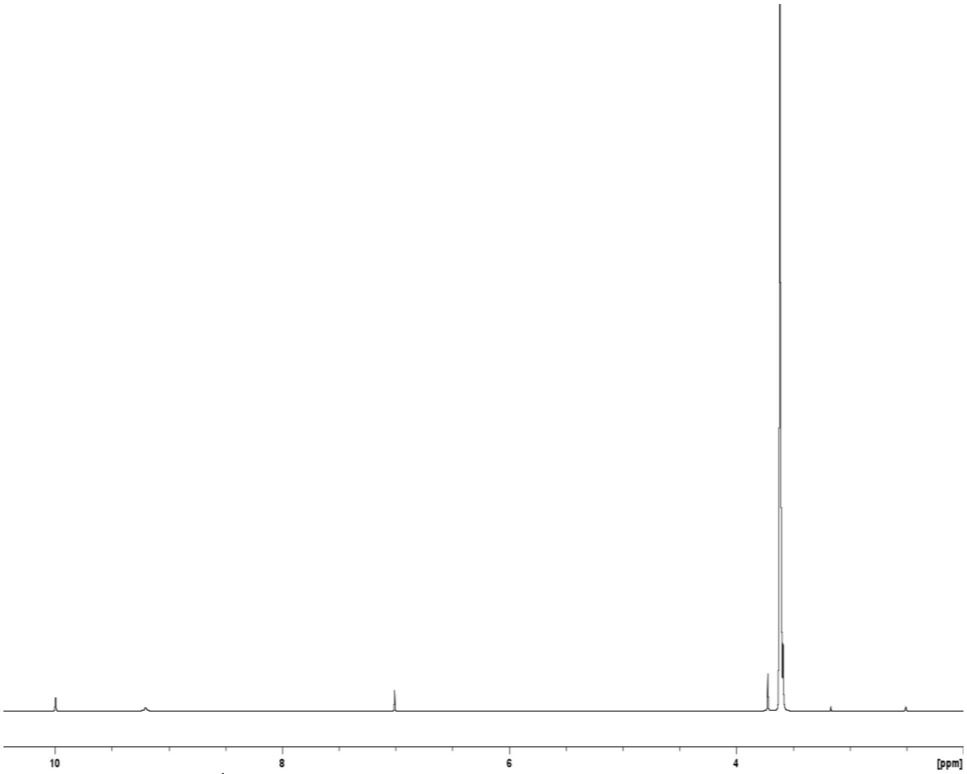

Figure S59: 1D ^{1}H-NMR spectrum of subereaphenol B (**17**) in DMSO-d_6, 303 K, 600 MHz.

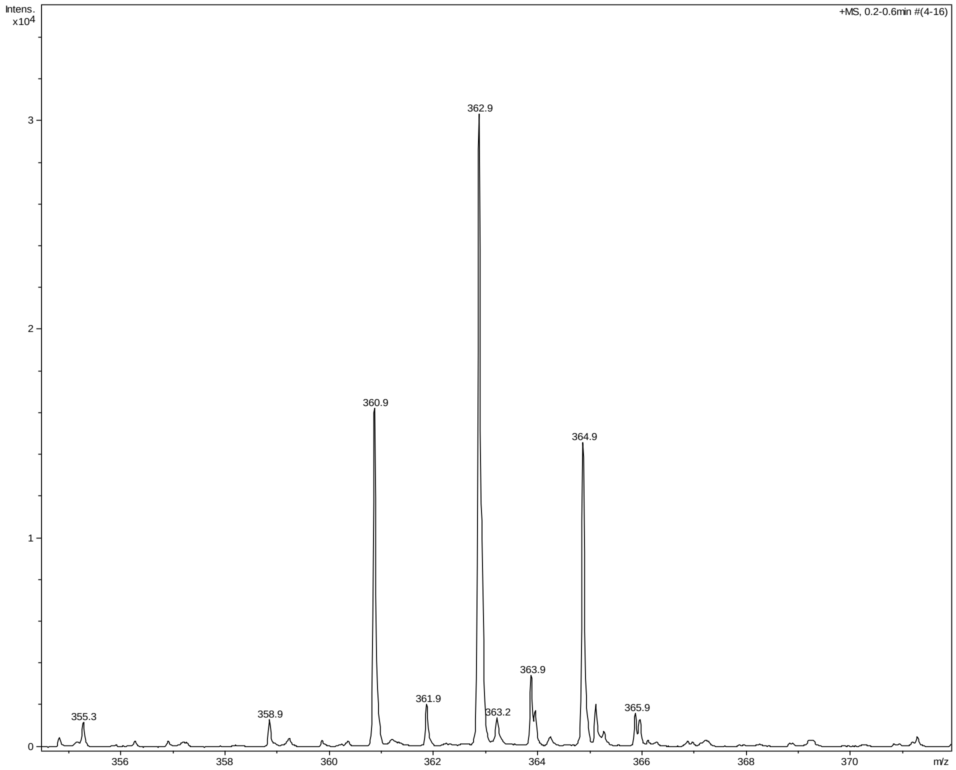

Figure S60: ESI–MS(+) spectral data of subereaphenol B (**17**).

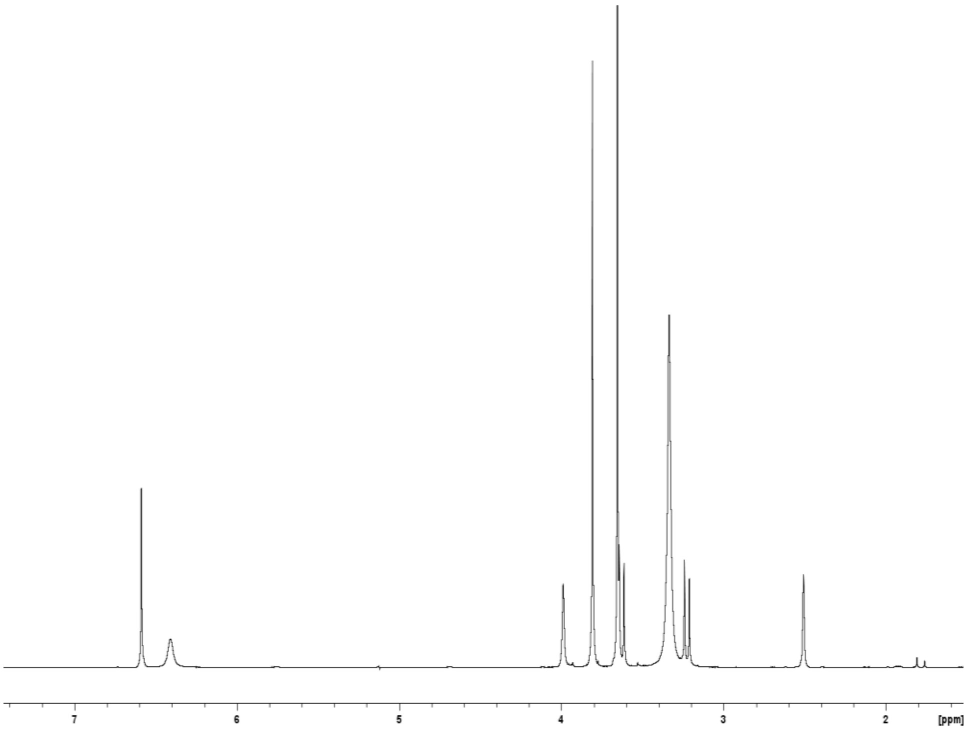

Figure S61: 1D ^{1}H-NMR spectrum of unnamed bromytyrosine (**18**) in DMSO-d_6, 303 K, 600 MHz.

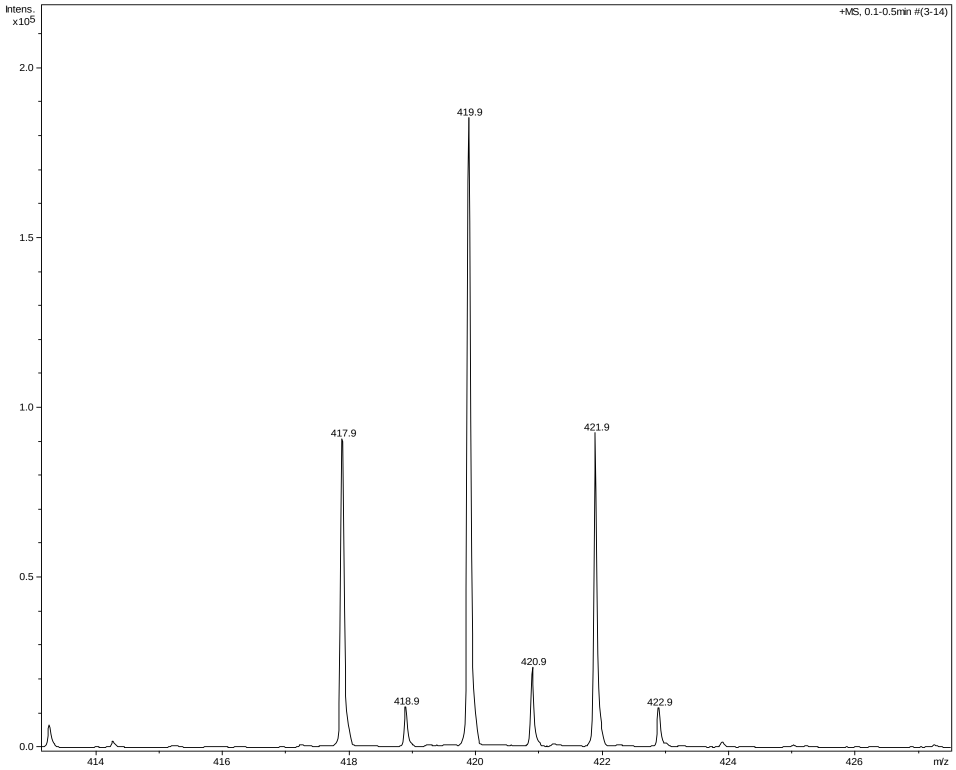

Figure S62: ESI–MS(+) spectral data of unnamed bromytyrosine (**18**).

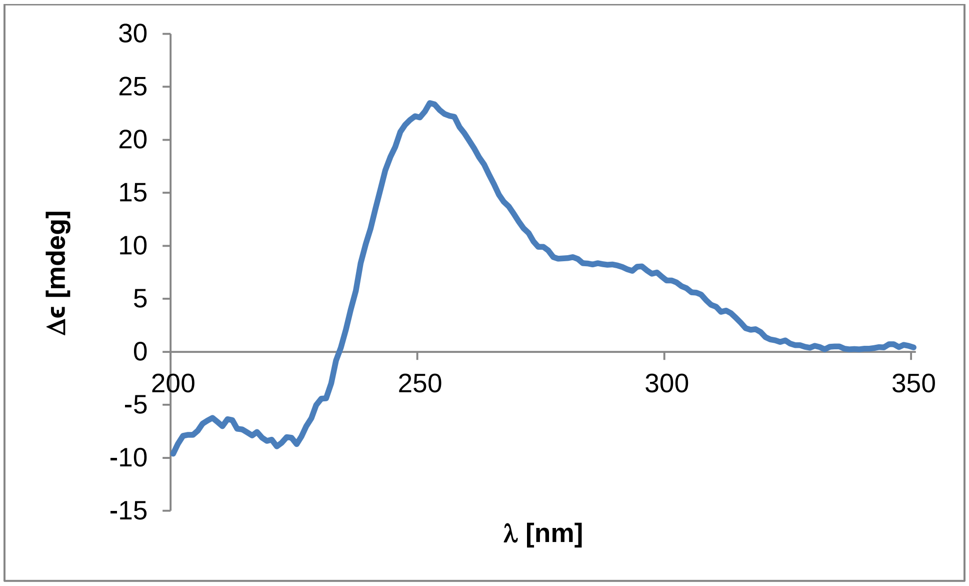

Figure S63: Circular dichroism spectrum of unnamed bromytyrosine (**18**) (c = 4.8 × 10^{-4} M, MeOH, 25 °C)
